# Safety and Tolerability of the Adeno-Associated Virus Vector, AAV6.2FF, Expressing a Monoclonal Antibody in Murine and Ovine Animal Models

**DOI:** 10.3390/biomedicines9091186

**Published:** 2021-09-09

**Authors:** Amira D. Rghei, Laura P. van Lieshout, Benjamin M. McLeod, Yanlong Pei, Jordyn A. Lopes, Nicole Zielinska, Enzo M. Baracuhy, Brenna A. Y. Stevens, Sylvia P. Thomas, Jacob G. E. Yates, Bryce M. Warner, Darwyn Kobasa, Hugues Fausther-Bovendo, Gary P. Kobinger, Khalil Karimi, Brad Thompson, Byram W. Bridle, Leonardo Susta, Sarah K. Wootton

**Affiliations:** 1 Department of Pathobiology, Ontario Veterinary College, University of Guelph, Guelph, ON N1G 2W1, Canada; arghei@uoguelph.ca (A.D.R.); bmcleo03@uoguelph.ca (B.M.M.); ypei@ovc.uoguelph.ca (Y.P.); jlopes@uoguelph.ca (J.A.L.); zielinsn@uoguelph.ca (N.Z.); ebaracuh@uoguelph.ca (E.M.B.); bsteve04@uoguelph.ca (B.A.Y.S.); sthoma13@uoguelph.ca (S.P.T.); jyates01@uoguelph.ca (J.G.E.Y.); kkarimi@uoguelph.ca (K.K.); bbridle@uoguelph.ca (B.W.B.); lsusta@uoguelph.ca (L.S.); 2 Avamab Pharma Inc., 120, 4838 Richard Road SW, Calgary, AB T3E 6L1, Canada; lauravan93@hotmail.com (L.P.v.L.); bt@kickshawventures.com (B.T.); 3 Zoonotic Diseases and Special Pathogens, Public Health Agency of Canada, Winnipeg, MB R3E 3R2, Canada; bryce.warner@canada.ca (B.M.W.); darwyn.kobasa@canada.ca (D.K.); 4 Département de Microbiologie-Infectiologie et D’immunologie, Université Laval, Quebec City, QC G1V 0A6, Canada; faustlbv@gmail.com (H.F.-B.); gary.kobinger@crchudequebec.ulaval.ca (G.P.K.)

**Keywords:** adeno-associated virus (AAV) vector, vectored immunoprophylaxis, monoclonal antibody, safety, tolerability, large animal model

## Abstract

Adeno-associated virus (AAV) vector mediated expression of therapeutic monoclonal antibodies is an alternative strategy to traditional vaccination to generate immunity in immunosuppressed or immunosenescent individuals. In this study, we vectorized a human monoclonal antibody (31C2) directed against the spike protein of SARS-CoV-2 and determined the safety profile of this AAV vector in mice and sheep as a large animal model. In both studies, plasma biochemical parameters and hematology were comparable to untreated controls. Except for mild myositis at the site of injection, none of the major organs revealed any signs of toxicity. AAV-mediated human IgG expression increased steadily throughout the 28-day study in sheep, resulting in peak concentrations of 21.4–46.7 µg/ mL, demonstrating practical scale up from rodent to large animal models. This alternative approach to immunity is worth further exploration after this demonstration of safety, tolerability, and scalability in a large animal model.

## 1. Introduction

Monoclonal antibody (mAb) therapies have become a popular strategy for combatting infectious diseases, especially those without licensed vaccines or effective therapeutic treatment [1,2,3]. Recently, there has been an increase in research into mAbs that provide protection against a wide variety of viruses and bacteria, including SARS-CoV-2 [1,4,5,6,7]. Though mAbs are effective as therapeutics, production of clinical grade mAbs for passive immunization is costly and the resulting immunity is short-lived [8]. An alternative approach to passive immunization is vectorized antibody expression, in which an adeno-associated virus (AAV) vector is used to transfer a mAb gene to host cells that subsequently produce and secrete antibody into the blood [9,10]. In this manner, antibody expression is continuously manufactured within treated patients, providing immunity for much extended periods compared to passive antibody transfer.Vectorized antibody expression has been highly effective at protecting animal models from various infectious diseases including HIV, Influenza virus, Ebola virus and *Clostridium difficile* [11,12,13,14].

AAV vectors provide sustained expression of therapeutic transgenes; however, the potential of the AAV capsid or transgene product resulting in toxicity is a potential safety concern. To the best of our knowledge, there are no published studies examining the toxicity of AAV vectorized antibody expression; however, there is an abundance of preclinical and clinical studies that underscore the safety and tolerability of AAV-based therapies [15,16]. In this study, we selected a triple mutant AAV6 capsid, AAV6.2FF, as our vector of choice, due to its superior ability to transduce both muscle and lung tissue, as shown in murine and hamster animal models [13,14,17]. Here, we use AAV6.2FF to evaluate the safety and tolerability of this approach through hematology, blood biochemistry and histopathology and the feasibility of vectorized antibody expression in murine and ovine animal models to reach potentially therapeutic plasma antibody concentrations. Sheep represent a large, outbred animal model that is more genetically diverse than mice, and thus may be more reflective of what could happen in humans administered AAV vectors expressing mAbs. We hypothesized that AAV6.2FF-vectorized expression of human mAb, 31C2 would lead to robust antibody expression and would not negatively impact the immune system and general health of both murine and ovine animal models when intramuscularly administered.

## 2. Materials and Methods

### 2.1. AAV Vector Production

The heavy and light chain variable region gene sequences for monoclonal antibody 31C2 (kindly provided by H Fausther-Bovendo and G Kobinger, Laval University) were expressed from a single gene expression cassette under the control of a CASI promoter to generate human IgG heavy chain and lambda light chain polypeptides separated by a furin-F2A self-cleaving peptide [18]. A woodchuck hepatitis virus post transcriptional regulatory element (WPRE) and SV40 polyadenylation signal were included in the vector and the entire expression cassette was flanked by AAV2 inverted terminal repeats to facilitate packaging. AAV production was carried out as previously described using adherent HEK293 cells and heparin affinity chromatography [19].

### 2.2. AAV Titration

AAV vector genome titers were determined by qPCR. Vector samples were pre-treated sequentially with DNase (Promega M6101, Madison, WI, USA) and proteinase K (Invitrogen LSAM2546 Carlsbad, CA, USA) and purified by Qiagen Blood and Tissue Kit (Qiagen 69504, Germantown, Maryland). DNA samples were analyzed by qPCR using a TaqMan primer and probe set against the human IgG heavy chain sequence: forward primer 5’-TGCAACGTGAATCACAAGC-3’, reverse primer 5’-GCATGTGTGAGTTTTGTCAC-3’ and probe 5’ FAM/CACCAAGGT/Zen/GGACAAGAAA GTTGAGCCC/3IABkFQ (Integrated DNA Technologies Coralville, IA, USA), Luna universal qPCR master mix (New England Biolabs M3003, Ipswich, MA, USA) and a LightCycler 480 (Roche Nutly, NJ, USA) thermal cycler.

### 2.3. Animal Experiments

All animal experiments were approved by the University of Guelph Animal Care Committee in accordance with Canadian Council on Animal Care (CCAC) guidelines. Five-week-old BALB/c mice were purchased from Jackson Labs (Bar Harbor, ME, USA) and allowed to acclimatize for one week prior to study commencement. All mouse groups contained equal numbers of male and female animals. Mice received an intramuscular injection of AAV vector diluted in 1 × phosphate buffered saline (PBS) pH 7.4 to a final volume of 40 µL, which was administered to the gastrocnemius muscle using a tuberculin syringe. In-life blood draws were completed by saphenous bleed using EDTA microvettes (Sarstedt Inc, Newton, NC, USA). At endpoint, mice were deeply anesthetized with isoflurane for terminal retro-orbital blood collection in non-heparinized micro-hematocrit capillary tubes (DWK Life Sciences, Rockwood, TN, USA) or euthanized by isoflurane overdose for BAL. 800 μL of PBS was administered to lungs using a shielded 20G catheter (BD, Franklin Lakes, NJ, USA) and withdrawn after 2 min. The BALF was centrifuged at 2000 rpm for two minutes to pellet debris and the supernatant was collected.

Newborn male Dorset lambs nursed colostrum, as well as supplemental bovine dried colostrum, from their mothers for 48 hours prior to being adapted to consume milk replacer from a bottle feeder for the remainder of this study. Lambs were administered AAV6.2FF-31C2 by weight at a dose of 5 × 10^12^ VG/ kg. Vector was diluted in 1X PBS to a volume of 1 mL and multiple injections were delivered to the rump muscle, separated by approximately three inches. Injection sites were marked with spray paint to enable tissue collection from the site of injection at necropsy. Weekly blood draws were completed by jugular bleed using the Vacutainer blood collection system (BD, Franklin Lakes, NJ, USA).

Multiple sets of 25 mg samples of sheep lung tissue were rinsed with PBS and collected in 1.5 mL flat bottom Sarstedt O-ring tube (Sarstedt Inc, Newton, NC, USA). 500 µL RIPA buffer containing protease inhibitor (Thermo Fisher, Waltham, MA, USA) was added to the tube along with one 1/4” ceramic bead (MP 6540424, Santa Ana, CA, USA) and homogenized on the Precellys 24 tissue homogenizer (Thermo Fisher, Waltham, MA, USA) at 6000 rpm for 2 × one-minute cycles. Homogenized samples were then centrifuged at 13,000 rpm for 20 min at 4 °C to pellet cell debris. Supernatant was collected for subsequent hIgG quantification.

### 2.4. Enzyme-Linked Immunosorbent Assays (ELISAs)

Commercially available ELISA kits for human IgG (Abcam 195215, Cambridge, MA, USA) murine IgG (Abcam 157719, Cambridge, MA, USA) and ovine IgG (Abcam 190546, Cambridge, MA, USA) were used to determine plasma immunoglobulin concentrations. Human IgG concentrations in lavage fluid was expressed as human IgG concentration per mL of lavage sample.

Anti-AAV6.2FF capsid antibodies in sheep serum were detected using half well 96 well plates (Corning, NY, USA) coated with 30 μL of 1 × 10^10^ VG/mL AAV6.2FF-Luciferase overnight at 4 °C. Plates were then washed three times using 0.2% PBS-Tween20 (PBS-T) and blocked using SuperBlock buffer (Fisher 37515, Waltham, MA, USA) for 30 min. Two-fold plasma dilutions beginning at 1:400 were incubated at 37 °C for 60 min and then washed three times with 0.1% PBS-T. Donkey anti-sheep HRP conjugated secondary antibody (Thermo Fisher, Waltham, MA, USA) was diluted to 1:5000, added to each well and incubated at 37 °C for 60 min. The plate was then washed three times with 0.2% PBS-T and incubated with TMB substrate (Pierce PI34021 Waltham, MA, USA) for 15 min at room temperature. Reciprocal titer was defined as the highest serial serum dilution that gave an OD650 value 2-fold greater than the mean of the negative control wells.

### 2.5. Hematology and Clinical Chemistry

Terminal murine EDTA whole blood samples were submitted to the Animal Health Lab (AHL) at the University of Guelph (Guelph, ON, Canada) for manual leukocyte differential and a custom clinical chemistry panel using a Cobas 6000. Ovine EDTA and heparin whole blood samples were submitted weekly to the AHL for comprehensive complete blood count using an AVIDA 2120 and custom clinical chemistry panel using a Cobas 6000. Ovine reference ranges from the University of Guelph Animal Health Lab and University California Davis Veterinary Medical Teaching Hospital were consulted for analysis.

### 2.6. Cytokine Analysis

Murine plasma samples from the day 7 and day 28 cohorts were submitted to EveTechnologies (Calgary, AB, Canada) for mouse cytokine analysis using the Luminex 200 system (Luminex, Austin, TX, USA). Ten cytokines were simultaneously measured in the samples using Eve Technologies’ Mouse Focused 10-Plex Discovery Assay® (MilliporeSigma, Burlington, MA, USA) according to the manufacturer’s protocol. Plasma samples were diluted 2-fold with PBS pH 7.5 prior to analysis. Assay sensitivities of these markers range from 0.4–10.9 pg/ mL for the 10-plex.

### 2.7. Histology and Immunohistochemistry

Tissue samples were fixed for 24 hours in 10% formalin, washed twice with PBS and stored in 70% ethanol. Fixed tissues were submitted to AHL for histology processing and H&E staining. Pathology interpretation was completed blinded by a board-certified anatomic pathologist. Additional slides were submitted to AHL for IHC staining using an antibody against human IgG (Abcam 181236, Cambridge, MA, USA) at a 1:100 dilution following antigen retrieval using citrate buffer pH 6 and blocking with Protein Block Serum-Free (DAKO Corporation, Hamburg, Germany). EnVision+ system- HRP anti-Rabbit labelled polymer was used as secondary antibody according to the manufacturer’s instruction. NovaRed was used for HRP detection and slides were counterstained using hematoxylin. IHC staining was automated using Dako Autostiner Plus (DAKO Corporation, Hamburg, Germany).

### 2.8. AAV Vector Biodistribution

Tissues samples were frozen at necropsy and DNA was extracted from 25 mg of tissue using a Blood and Tissue Kit (Qiagen 69504 Germantown, MD, USA) and vector genome copy number was determined by qPCR as described above. DNA concentration was determined using Nanodrop and the vector genome copy number was normalized to the total genomic DNA concentration.

### 2.9. Statistics

Graphpad Prism 9 software (San Diego, CA, USA) was used to generate all statistical analyses. Mean and standard deviation are displayed on all graphs with the exception of the data from the lamb study in which each animal is plotted individually. One-way analysis of variance was used to evaluate the differences within cohorts. Tukey’s multiple comparison test was used as a post-test. An unpaired One-way ANOVA was used to compare the mouse weights over time. *p* values of < 0.05 was considered significant.

## 3. Results

### 3.1. Confirmation of AAV6.2FF-31C2 Vector Expression

**Figure 1:**
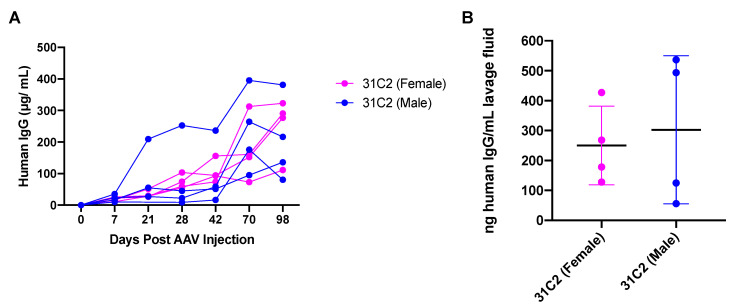
Evaluation of vectorized 31C2 monoclonal antibody. (**A**) 6-week-old male and female BALB/c mice (*n* = 4) were administered 2 × 10^10^ VG of AAV-mAb and plasma was monitored for hIgG expression over a period of 14 weeks. (**B**) BALF was collected at endpoint and hIgG was quantified. Data are represented as the mean ± standard deviation.

Heavy and light chain variable region sequences for 31C2 were kindly provided by collaborators working to isolate and characterize mAbs from COVID-19 patient samples. The AAV vector genome expressing 31C2 as human IgG1 under the control of a strong ubiquitous promoter was packaged using the AAV6.2FF capsid. Groups of male and female (*n* = 4) BALB/c mice were injected intramuscularly (IM) with 2 × 10^10^ vector genomes (VG) of AAV6.2FF-31C2. Blood draws occurred on day 0, 7, 21, 28, 42, 70 and 98 post AAV administration and plasma was analyzed for human IgG (hIgG) concentration to evaluate transgene expression (Figure 1A). 

At approximately 14 weeks post vector administration, these mice were sacrificed, and bronchoalveolar lavages (BAL) were performed and hIgG in the lavage fluid was quantified (Figure 1B). BAL fluid (BALF) trended similarly to plasma hIgG concentrations; expressing 31C2 had an average of 121.6 nanograms (ng)/mL lavage fluid, demonstrating robust plasma concentrations are required for antibody diffusion into the lung mucosal surface. Differences in the hIgG levels in both the plasma and BALF varied based on sex differences and due to variability during IM administration of the vector and efficiency of BALF collection. 

### 3.2. Safety and Tolerability of AAV6.2FF-31C2 in a Murine Model

A mouse study was designed to investigate the safety and tolerability of the AAV6.2FF-31C2 vector administered to the muscle. The study design utilized three cohorts to evaluate endpoints at 7-, 28- and 56-days post AAV administration at a low (1 × 10^11^ VG), mid (2 × 10^11^ VG) and high (6 × 10^11^ VG) doses (*n* = 6; 3 males and 3 females) (Table 1). Mice were dosed based on a fixed vector genome concentration; however, when adjusted for the weight the average dose per kilogram (kg) was an average on 4.7 × 10^12^, 9.4 × 10^12^ and 2.9 × 10^13^ VG/ kg for the low, mid and high dose groups, respectively (Figure S1). Due to the limited amount of blood that can be taken at one time point, only terminal blood draws were taken from the mice at endpoint for analysis.

**Figure 2:**
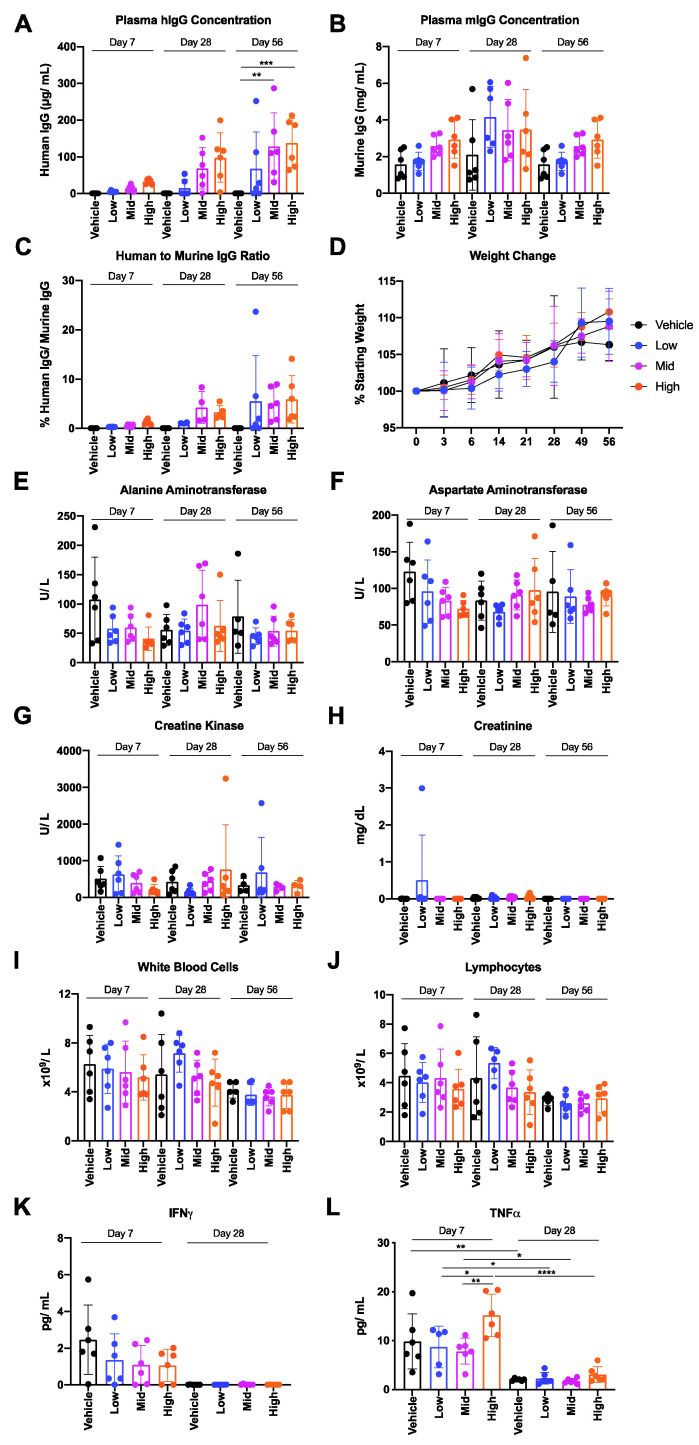
Findings of the murine AAV6.2FF-31C2 safety and tolerability study. 6-week-old male and female BALB/c mice (*n* = 6; equal male and female mice per group) were administered either a low (1 × 10^11^ VG), mid (2 × 10^11^ VG) or high (6 × 10^11^ VG) dose and sacrificed either 7-, 28- or 56-days post AAV administration. Terminal blood samples were collected at endpoint and plasma was analyzed for (**A**) hIgG concentration, (**B**) mIgG concentration and (**C**) the ratio of hIgG to mIgG is shown. (**D**) Mice were weighted on a weekly/ biweekly schedule throughout the in-life phase of the study. Plasma and whole blood samples were analyzed for (**E**) alanine aminotransferase, (**F**) aspartate aminotransferase, (**G**) creatine kinase, (**H**) creatinine, (**I**) white blood cells, (**J**) lymphocytes, IFNγ (**K**) and TNFα (**L**). A one-way ANOVA was used for analysis. Data are represented as the mean ± standard deviation. * *p* < 0.05, ** *p* < 0.01, *** *p* < 0.001 and **** *p* < 0.0001.

A dose dependent increase in hIgG transgene in murine plasma was observed between the low, mid and high dose cohorts across the three time points (days 7, 28 and 56) (Figure 2A). hIgG expression was highest 56 days post vector delivery resulting in average plasma hIgG concentrations of 67.9, 127.7 and 137.8 μg/ mL for groups administered with low, mid and high doses, respectively. Interestingly, a minimal increase in plasma 31C2 was observed between groups administered with mid-dose and high-dose, despite a 3-fold increase in VG dose administered; however, it is possible that the high dose of AAV saturated the number of muscle cells in the leg susceptible to transduction. hIgG expression did not significantly alter murine IgG (mIgG) concentrations compared to vehicle treated control groups (Figure 2B). The average mIgG across day 7, 28 and 56 cohorts were 2.2, 3.3 and 2.3 mg/ mL, respectively (Figure 2B). The proportion of hIgG to mIgG increased from an average of 0.28–1.20% in the day 7 cohort to 0.72–4.25% in the day 28 cohort to 3.52–5.49% in the day 56 cohort (Figure 2B). All mice had a maximum of 9% of hIgG present except for two outliers in groups 9 (Day 56-low) and 11 (Day 56-high), each with high hIgG concentrations 212.4–251.9 μg/ mL and low mIgG concentrations 1.1–1.5 mg/mL, resulting in high hIgG proportions of 14.1% and 24% of mIgG. All groups gained weight similarly to the vehicle controls throughout the course of the study, indicating general health was not negatively impacted by AAV6.2FF transfection and AAV6.2FF-mediated mAb expression (Figure 2D). Mice were terminally bled at endpoint and samples were submitted for leukocyte differential, clinical biochemistry and an inflammatory cytokine panel. Plasma alanine aminotransferase, aspartate aminotransferase, creatine kinase and creatinine concentrations were not significantly elevated across treatment groups for any cohort, indicating that liver and muscle damage was not present (Figure 2E–H). Serum albumin, globulin, total protein and urea concentrations were also evaluated (Figure S2).

Lymphocyte differential demonstrated no significant trends in total white blood cells, lymphocytes, neutrophils, monocytes or eosinophils between groups or cohorts, indicating a lack of lymphocyte based immune response captured within the time points of this study (Figure 2I,J and Figure S3). IFNγ and TNFα were both elevated in all treatment groups, including mock groups, at day 7, but the response subsided by day 28, demonstrating that this response was acute and resolved quickly (Figure 2K,L). IL-1B, IL-2, IL-4, IL-6, IL-10, IL-12p70, MCP-1 and GM-CSF concentrations were also evaluated but the trend for IFNγ and TNFα was not observed for other cytokines (Figure S4). The day seven increase was only significant for TNFα and none of the observed concentrations for this panel of cytokines are indicative of a strong inflammatory response. Nevertheless, these data demonstrate there was a slight elevation in select cytokines that resolved by day 28. Overall, this mouse study revealed no significant changes in blood chemistry or hematological parameters as a result of vectorized 31C2 expression.

Tissues from the high dose groups, euthanized 56 days following AAV-31C2 injection, were used for biodistribution analysis. Unfortunately, due to the small size of mouse gastrocnemius muscles, the entire sample was submitted for histology and was therefore not included here. While AAV vector genomes were detected in all tissues examined, only in the kidney and liver was there a significant, dose dependent increase in AAV genome copy numbers (Figure S5).

### 3.3. Safety and Tolerability of AAV6.2FF-31C2 in a Pediatric Ovine (Sheep) Model

**Figure 3:**
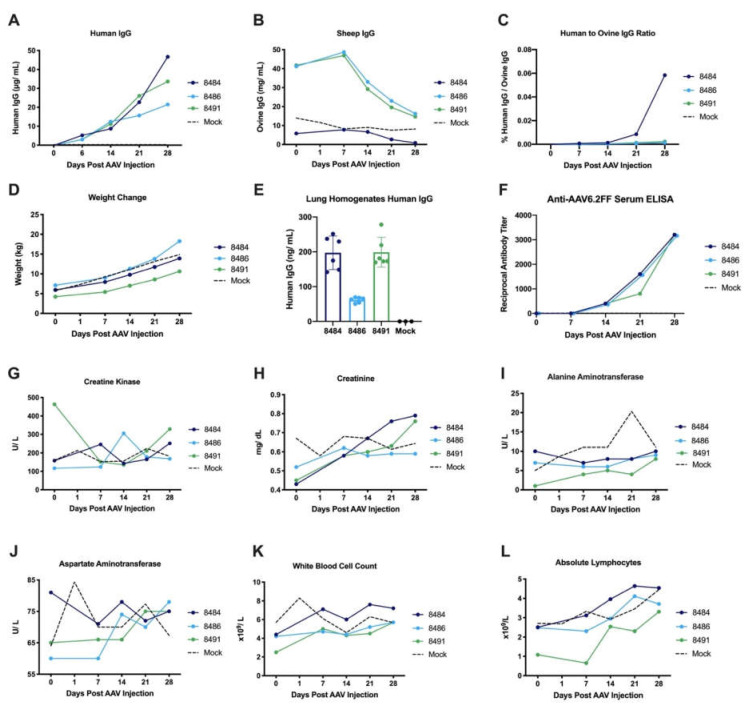
Findings of the ovine AAV6.2FF-31C2 feasibility study. Three 2-week-old lambs were administered 5x10^12^ VG/ kg of AAV6.2FF-31C2 by IM injections to the rump. Aged-matched controls (*n* = 3) were administered PBS IM. Weekly blood samples were collected and analyzed for plasma (**A**) hIgG concentration, (**B**) oIgG concentration and (**C**) the calculated ratio of hIgG to oIgG is displayed. (**D**) Sheep were weighted weekly throughout the in-life phase of the study. (**E**) Lung homogenates at endpoint (two sample sites per animal) were analyzed for hIgG concentration. (**F**) Anti-AAV6.2FF capsid antibodies were monitored weekly. Plasma and whole blood samples were analyzed for (**G**) creatine kinase, (**H**) creatinine, (**I**) alanine aminotransferase, (**J**) aspartate aminotransferase, (**K**) white blood cells and (**L**) absolute lymphocytes. The black dotted line represents the average value for the three control sheep.

An additional goal of the sheep study was to evaluate the feasibility of the AAV-mAb approach in a large, outbred animal model to support the data previously generated using this platform in rodent models. Three two-week-old male lambs were administered 5 × 10^12^ VG/ kg of AAV6.2FF-31C2 by IM injections to the rump. An additional three two-week-old male lambs served as controls. The lambs weighed 4.22, 5.94 and 7.12 kg at the time of vector administration and required two to three injections to administer the total volume of AAV vector. Lambs were evaluated for a four week in-life phase prior to endpoint to evaluate pathology. Blood was drawn on a weekly basis to evaluate transgene expression and plasma hIgG concentrations increased steadily throughout the study, peaking at 21.4, 33.7 and 46.7 μg/ mL at the final time point (Figure 3A). Ovine IgG (oIgG) concentrations were also evaluated to determine the ratio of hIgG to oIgG (Figure 3B,C). The newborn lambs in the study received colostrum and were removed from the mother ewes after approximately 48 hours; at which point they were adapted to bottle milk supplemented with bovine dried colostrum. Therefore, the higher level of oIgG in Sheep 8486 and 8491 could be attributed to these animals having received more colostrum than the other sheep in this study.

The hIgG transgene represents much less than 0.1% of the total oIgG due to the larger blood volume compared to mice. Lambs gained weight as expected throughout the in-life phase on the study, more than doubling in weight over 28 days (Figure 3D). Interestingly, the level of hIgG increased despite this rapid weight increase, demonstrating efficient expression of the transgene in this sheep model.

In the lung tissue homogenates, Sheep 8484 and 8491 had higher levels of hIgG than 8486, reflecting the trend of hIgG levels observed in the plasma, similar to the pattern observed previously in murine lung lavages (Figure 3E). These results confirm that delivery of AAV6.2FF-31C2 to the muscle results in antibody diffusion to the lung, the primary organ involved in respiratory infections. Low levels of anti-AAV6.2FF capsid antibodies were detected in each of the sheep, which show an upward trend over the 28-day sampling period (Figure 3F).

Data was collected to assess safety and tolerability of this large animal model feasibility study. Plasma creatine kinase, alanine aminotransferase, and aspartate amino transferase did not demonstrate any notable increases above the reference ranges and in comparison, to sham control sheep (Figure 3G–J). Similarly, lactate dehydrogenase, urea, glucose, bilirubin, total protein, globulin and albumin and plasma alkaline phosphate concentrations remained steady or within the references ranges throughout the study and in comparison, to sham control sheep (Figure S6). White blood cell and lymphocyte counts trended slightly upwards but remained within the ovine reference range (Figure 3K,L). Red blood cell count, hemoglobin and hemocrit concentrations as well as platelets, neutrophil, monocyte, eosinophil and basophil counts were also monitored with few notable deviations from controls (Figure S7). In the AAV6.2FF-31C2 group, there was a spike in neutrophils in two out of three sheep on day seven that returned to baseline by day 14; however, the values were within the upper range of the reference interval.

To better understand the biodistribution of AAV6.2FF-31C2 following IM injections in tissues other than the vector injected muscle, tissues collected from sheep euthanized 28 days post AAV6.2FF-31C2 injection were analyzed for AAV vector genome biodistribution (Figure S8). Only in the kidney, heart, brain, and spleen of sheep # 8491 were AAV vector genomes above background levels.

### 3.4. Lack of Pathology Observed in Murine and Ovine Models Following Intramuscular AAV-mAb Administration

**Figure 4:**
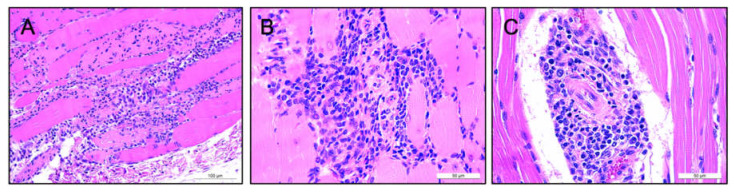
Comparison of striated murine and ovine muscle tissue using H&E staining. Histopathological images of inflamed murine muscle tissue 28 days post IM administered of 5 × 10^12^ VG/kg of AAV-31C2 at 20× (**A**) and 40× (**B**), showing lymphocytes and macrophages in the endomysium surrounding numerous myofibers. Straited muscle tissue from Sheep #8486 28 days post IM administered of 5 × 10^12^ VG/kg of AAV-31C2 is seen at 40× (**C**). Scattered lymphocytes, plasma cells and rare macrophages were present surrounding myofibers, with accumulation seen in the endomysium and medium-size vessels.

We examined murine tissues histologically to determine whether AAV-31C2 led to any adverse effects in the lung, heart, brain, kidney, spleen, liver or gastrocnemius muscle. Mild inflammation of the muscle at the site of injection was observed in all groups, with the exception of the vehicle only groups, indicating some inflammation was induced by the vector rather than from the injection alone. Only four mice exhibited inflammation extending to > 50% of the muscle tissue, including mice from low and high dose groups (Figure 4A,B) (Supplemental Table S1). These findings were attributed to the IM route of AAV injections. All liver tissue sections showed mild lipid-type vacuolations mainly in centrilobular spaces, not indicative of pathological origin. The brain and kidney demonstrated normal histology, with no indication of toxicity. Minor irregularities of other organs included mild mineralization and scattered lymphocytes in the epicardium, and extramedullary hematopoiesis (EMH) in the lung. No evidence of lesions or other pathological changes was observed in these organs; therefore, these observations were deemed incidental findings.

Tissues from sheep administered AAV6.2FF-31C2 were similarly examined to determine if any toxicity was attributed to the vector. Mild inflammation of striated muscle tissue was observed in the node nerve, intestines, lymph node, thymus, skin and muscle. Only muscle tissue found directly at the site of injection and in intestine tissue of Sheep 8486 was considered greatly affected, although all striated muscle was deemed clinically insignificant (Figure 4C). These findings are consistent with the lack of gross lesions and other clinical observations.

Sheep 8484 showed mild inflammation of the epicardium, while all animals showed EMH in the liver and the presence of mild interstitial lymphocytes in the kidney. These findings were considered incidental and can be attributed to hematopoiesis and lymphopoiesis seen in lambs. None of the tissues sampled from the large intestine, small intestine, abomasum, lymph node, brain, spleen, lung, testis, bladder, pancreas, thymus, liver, kidney, bone marrow, thyroid, parathyroid, heart, skin, and muscle tissue showed any signs of vector-related toxicities. Overall, none of the organs observed in both the murine and ovine models showed any signs of adverse clinical effects linked to AAV6.2FF-31C2 treatment.

### 3.5. Human IgG Is Expressed in Murine and Ovine Muscle

**Figure 5:**
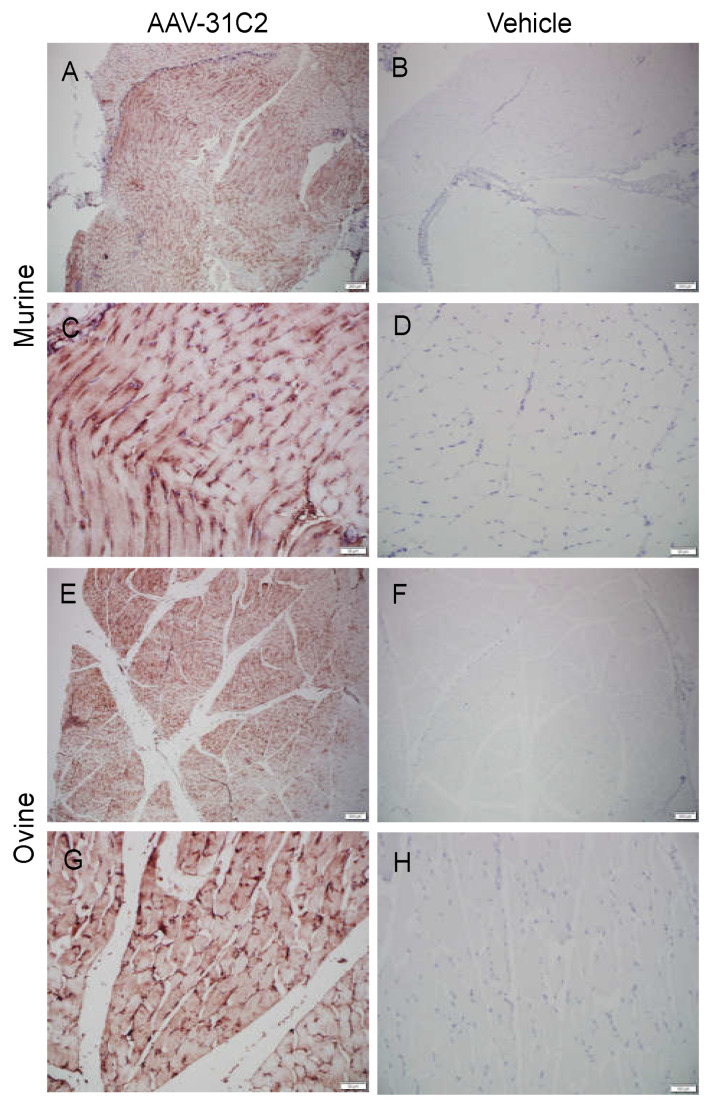
Immunohistochemical staining of human IgG in murine and ovine muscle. Stained murine muscle tissue from a mouse intramuscularly injected with 5 × 10^12^ VG/kg AAV6.2FF-31C2 28 days prior at (**A**) 4× and (**C**) 20× and negative control murine muscle receiving vehicle only at (B) 4× and (**D**) 20× Stained ovine muscle tissue from sheep #8486 28 days post injection with 5 × 10^12^ VG/kg AAV6.2FF-31C2 at (**E**) 4× and (**G**) 20× and healthy negative control tissue at (**F**) 4× and (**H**) 20×.

While quantification of hIgG in the plasma demonstrates successful vectorized antibody expression, we sought to confirm expression at the site of injection. Muscle samples from murine and ovine subjects were prepared for histology and immunohistochemical staining of hIgG was conducted. Widespread hIgG staining was observed in the skeletal muscle surrounding the site of IM AAV6.2FF-31C2 administration for both species (Figure 5).

## 4. Discussion

Vectorized long-term expression of therapeutic antibodies provides a promising alternative to traditional immunizations. Classical antigen-based vaccines require a robust endogenous immune response to provide immunity and the age-related decline in the immune function in those age 65 or older, as well as immunocompromised individuals, leads to increased vulnerability to infection and a decrease in vaccine responsiveness [20,21]. Studies show the effectiveness of influenza vaccines are reduced as much as 50% in those 65 years of age or older compared to young adults [22,23,24]. Here we describe safety, tolerability and feasibility of utilizing the AAV6.2FF capsid to express SARS-CoV-2 mAb 31C2 in both murine and ovine animal models.

All cohorts of mice displayed tolerability to increasing dosages of AAV6.2FF-31C2, with no notable pathology. Any alterations in cytokine levels in the mouse tox study were resolved after the day 7 time point; however, it is probable that the peak of the cytokine response occurred well before the first cohort endpoint. Regarding future studies, cytokine responses at early time points (e.g., 2 to 12 hours post-AAV administration) would shed light on the extent to which IM administration of AAV6.2FF activates the innate immune response. Elevated levels of IFNγ and TNFα were detected in the vehicle only treated groups of mice as well as in the mice receiving AAV, suggesting that the injection itself might have induced this response. Analysis of the biodistribution of AAV in murine tissues revealed a statistically significant dose-dependent increase in AAV vector genomes in the liver and kidney, which is not surprising as both are organs with high blood flow. However, higher levels of AAV genomes in the liver and kidney did not have an impact on the tolerability of AAV.

Previous non-human primate (NHP) studies have shown that AAV expression of monoclonal antibodies against simian immunodeficiency virus (SIV) is an effective method for prolonged antibody-based immunity to challenge [25,26]. Stable vectorized expression of the anti-SIV monoclonal antibody 5L7 has been maintained in NHPs at 240-350 μg/ mL for over 6 years [25]. While our study was terminated after 4 weeks to evaluate pathology, long term expression studies in the sheep model are currently ongoing. AAV8-mediated delivery of two anti-SIV mAbs directed against the Env protein resulted in peak plasma concentrations of 21.2 and 8.6 μg/mL while providing protection from weekly limiting dose intrarectal SIV challenges [27]. In our sheep study, we observed a steady weekly increase in 31C2, which peaked as high as 46.7 μg/mL after 28 days, despite the rapid > 2.3-fold weight gain post vector administration. Although NHPs are an ideal large animal model due to their similarity to humans, adult macaques generally weigh less than 20 kg, a mass lambs reached in a matter of weeks with adult sheep weighing more than 80 kg, which is a more clinically relevant model in terms of human size, blood volume and lung physiology. Continued monitoring of the sheep to determine longevity of AAV-mediated expression of 31C2 in the plasma as the lambs grew to full-sized adults would provide insight into how well AAV6.2FF could express mAbs in an adult human.

One of the limitations of this study was the fact that we were unable to perform bronchiolar lavages on the sheep. While lung tissue from the sheep was retrieved and analyzed for hIgG expression, quantification of hIgG in BALF would be very informative and would have allowed us to compare the proportion of hIgG in serum vs. lung fluid, like we did in the mice.

To the best of our knowledge, this is the first published data regarding the safety and tolerability of AAV-mediated antibody expression in vivo. However, there are numerous reports evaluating AAV-based therapies for other applications, demonstrating the strong safety profile of AAV vectors [28,29,30]. In mice, mild and acute liver pathology has been observed following systemic delivery of AAV8 and AAV9 gene therapy vectors at 6 × 10^13^ and 1 × 10^14^ VG/ kg; however, these were toxicology studies designed to understand the potential toxicities associated with high clinical doses while this study focused on vector tolerability at a functional dose [31,32].

Overall, our results exhibited a lack of significant alterations in serum biochemistry or histopathological findings in both murine and ovine studies, suggesting AAV vectorized antibody expression is a safe approach to generate immunity. Given the absence of notable toxicity findings in combination with the demonstration of large animal model feasibility, further development of the AAV6.2FF-mAb platform is warranted, including the evaluation of anti-drug antibody (ADA) responses and efficacy studies in non-human primate challenge models.

## Figures and Tables

**Table 1:** Mouse safety and tolerability study design.

Group	Cohort	Treatment	Dose (VG)	Endpoint	Number of Animals ^2^
1	Day 7-low	AAV6.2FF-31C2	1 × 10^11^	Day 7	*n* = 6
2	Day 7-mid	AAV6.2FF-31C2	2 × 10^11^	Day 7	*n* = 6
3	Day 7-high	AAV6.2FF-31C2	6 × 10^11^	Day 7	*n* = 6
4	Day 7-mock	Vehicle only ^1^	-	Day 7	*n* = 6
5	Day 28-low	AAV6.2FF-31C2	1 × 10^11^	Day 28	*n* = 6
6	Day 28-mid	AAV6.2FF-31C2	2 × 10^11^	Day 28	*n* = 6
7	Day 28-high	AAV6.2FF-31C2	6 × 10^11^	Day 28	*n* = 6
8	Day 28-mock	Vehicle only ^1^	-	Day 28	*n* = 6
9	Day 56-low	AAV6.2FF-31C2	1 × 10^11^	Day 56	*n* = 6
10	Day 56-mid	AAV6.2FF-31C2	2 × 10^11^	Day 56	*n* = 6
11	Day 56-high	AAV6.2FF-31C2	6 × 10^11^	Day 56	*n* = 6
12	Day 56-mock	Vehicle only ^1^	-	Day 56	*n* = 6

^1^ 1 × PBS-phosphate buffered saline was used as the vehicle, ^2^ 3 male and 3 female mice per group, - Not applicable.

## Data Availability

Data is contained within the article or supplementary material.

## Supplementary Materials

The following are available online at https://www.mdpi.com/article/10.3390/biomedicines9091186/s1, Figure S1: Calculated murine AAV6.2FF-31C2 vector genome dose by weight, Figure S2: Additional murine clinical chemistry, Figure S3: Additional murine hematology, Figure S4: Additional murine cytokine profiles, Figure S5: Biodistribution of AAV in Murine Tissues following IM administration of AAV6.2FF-31C2, Figure S6: Additional ovine clinical chemistry, Figure S7: Additional ovine hematology, Figure S8: Biodistribution of AAV in Ovine Tissues following IM administration of AAV6.2FF-31C2, Table S1: Mouse H&E Grading, Table S2: Sheep H&E Grading.
