# Supplementary material for: Safety and Tolerability of the Adeno-Associated Virus Vector, AAV6.2FF, Expressing a Monoclonal Antibody in Murine and Ovine Animal Models
Source: Biomedicines. 2021 Sep 9;9(9):1186. doi: 10.3390/biomedicines9091186 (PMC8464737; doi:10.3390/biomedicines9091186)
Supplement: Supplementary file 1 [file biomedicines-09-01186-s001.zip › biomedicines-1362655-supplementary.pdf]

biomedicines

Safety and tolerability of the adeno-associated virus vector, AAV6.2FF, expressing a monoclonal antibody in murine and ovine animal models

Supplementary Material

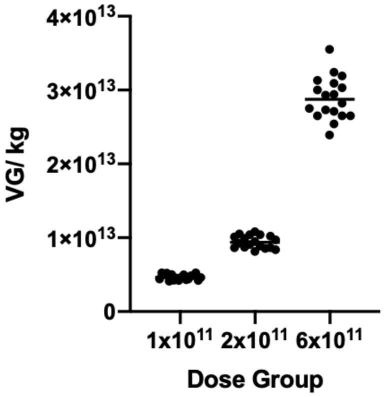

Supplemental Figure S1. Calculated murine AAV6.2FF-31C2 vector genome dose by weight. Vector genome dose was nor-malized to individual mouse weights on the day of AAV6.2FF-31C2 administration to determine the calculated dose by weight for comparison with the lamb feasibility dosing scheme.

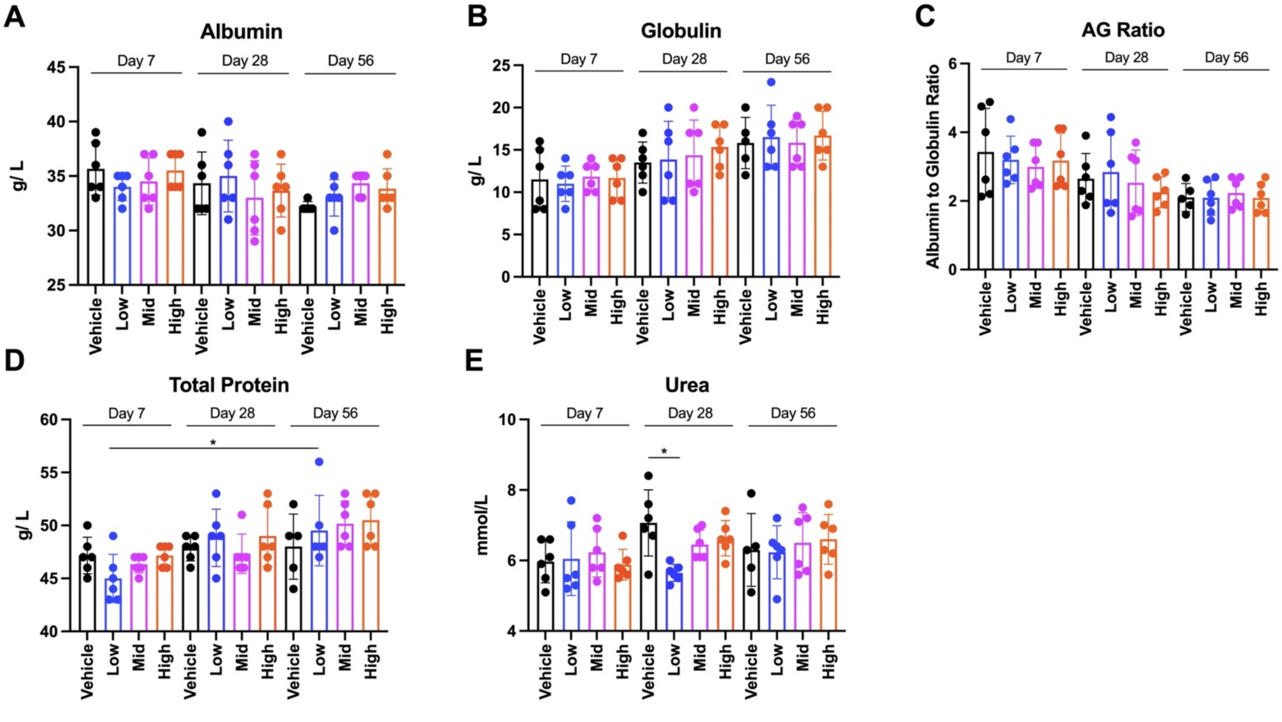

Supplemental Figure S2. Additional murine clinical chemistry. Plasma concentrations of (A) Albumin, (B) globulin, (C) the albumin to globulin ratio, (D) total protein and (E) urea. *p<0.05.

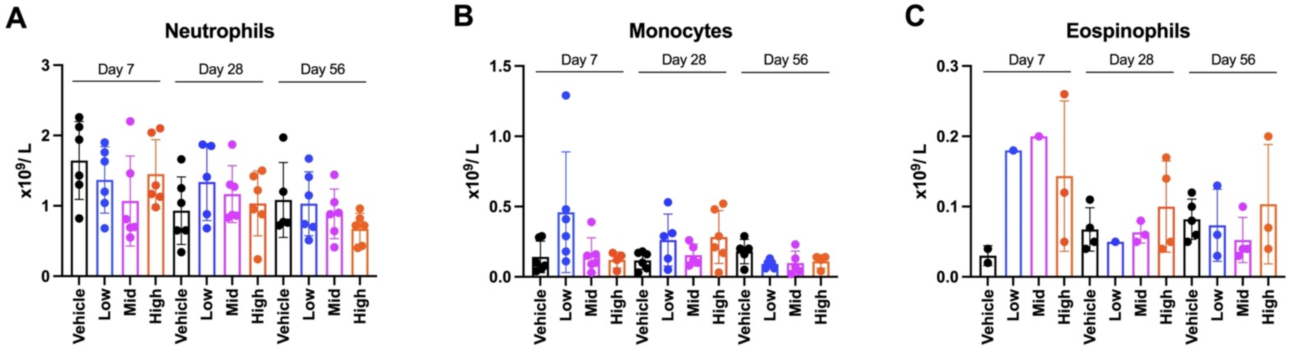

Supplemental Figure S3. Additional murine hematology. (A) Neutrophil, (B) monocyte and (C) eosinophil counts.

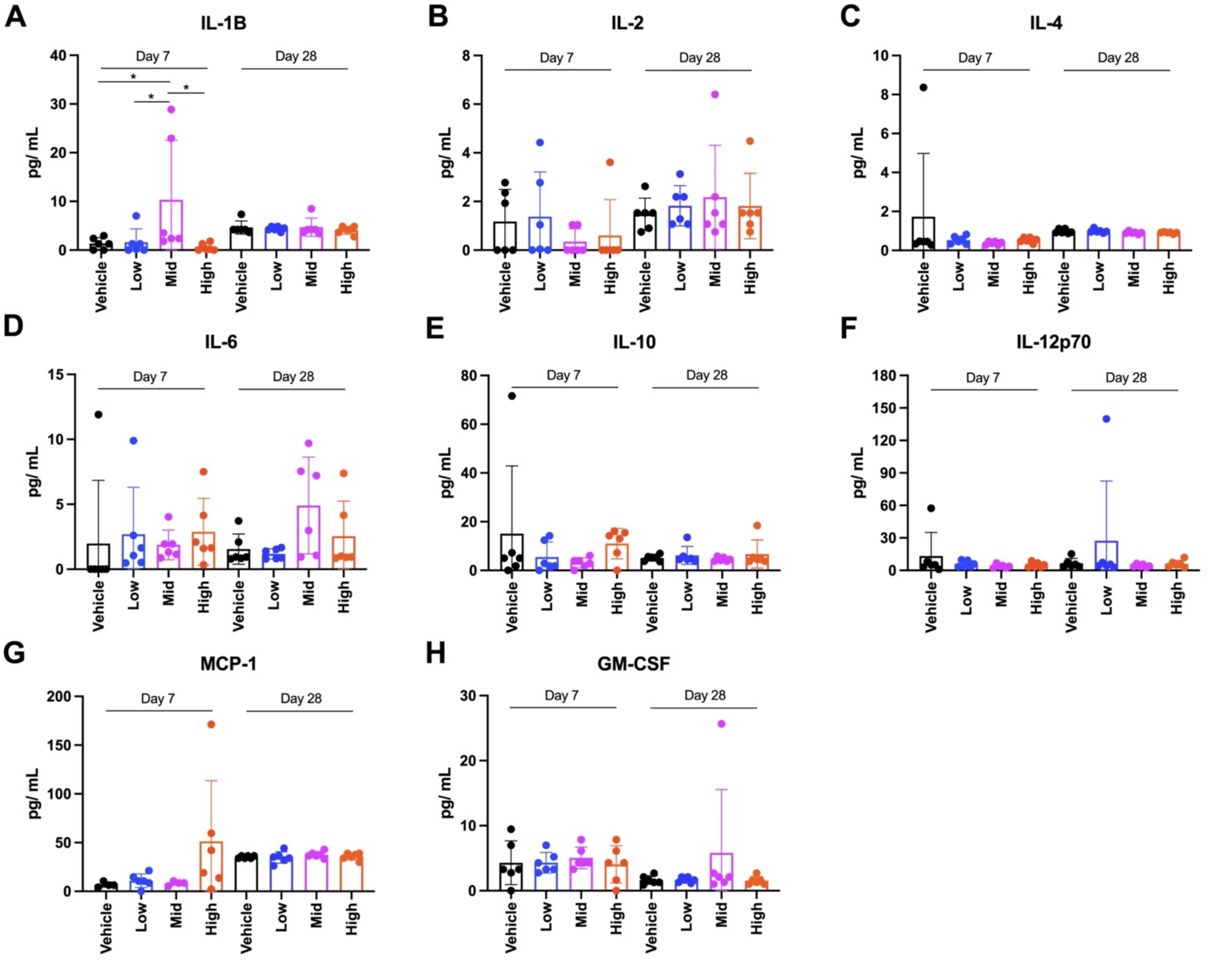

Supplemental Figure S4. Additional murine cytokine profiles. Plasma concentrations of (A) IL-1B, (B) IL-2, (C) IL-4, (D) IL-6, (E) IL-10, (F) IL-12p70, (G) macrophage chemoattractant protein -1 and (H) granulocyte-macrophage colony-stimulating fac-tor. *$p<0.05$.

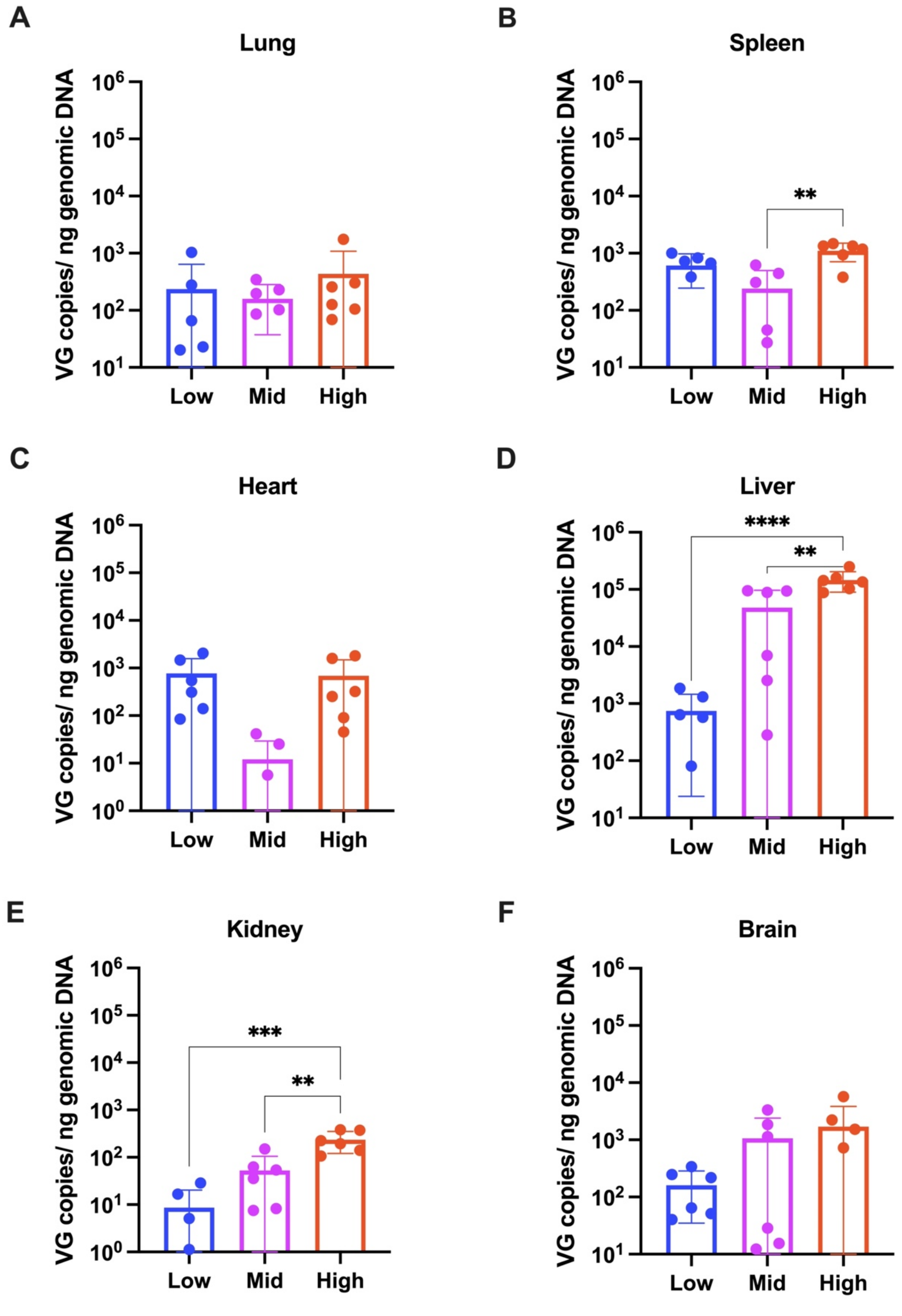

Supplemental Figure S5. Biodistribution of AAV in Murine Tissues following IM administration of AAV6.2FF-31C2. AAV vector genomes from (A) kidney, (B) liver, (C) lung, (D) heart, (E) brain and (F) spleen tissue samples were quantified by qPCR and shown as VG copies/ng genomic DNA after average background values for each tissue group from PBS injected mice were subtracted. Data are represented as the mean ± standard deviation using a one-way ANOVA. *p<0.05, **p<0.01, ***p<0.001 and ****p<0.0001.

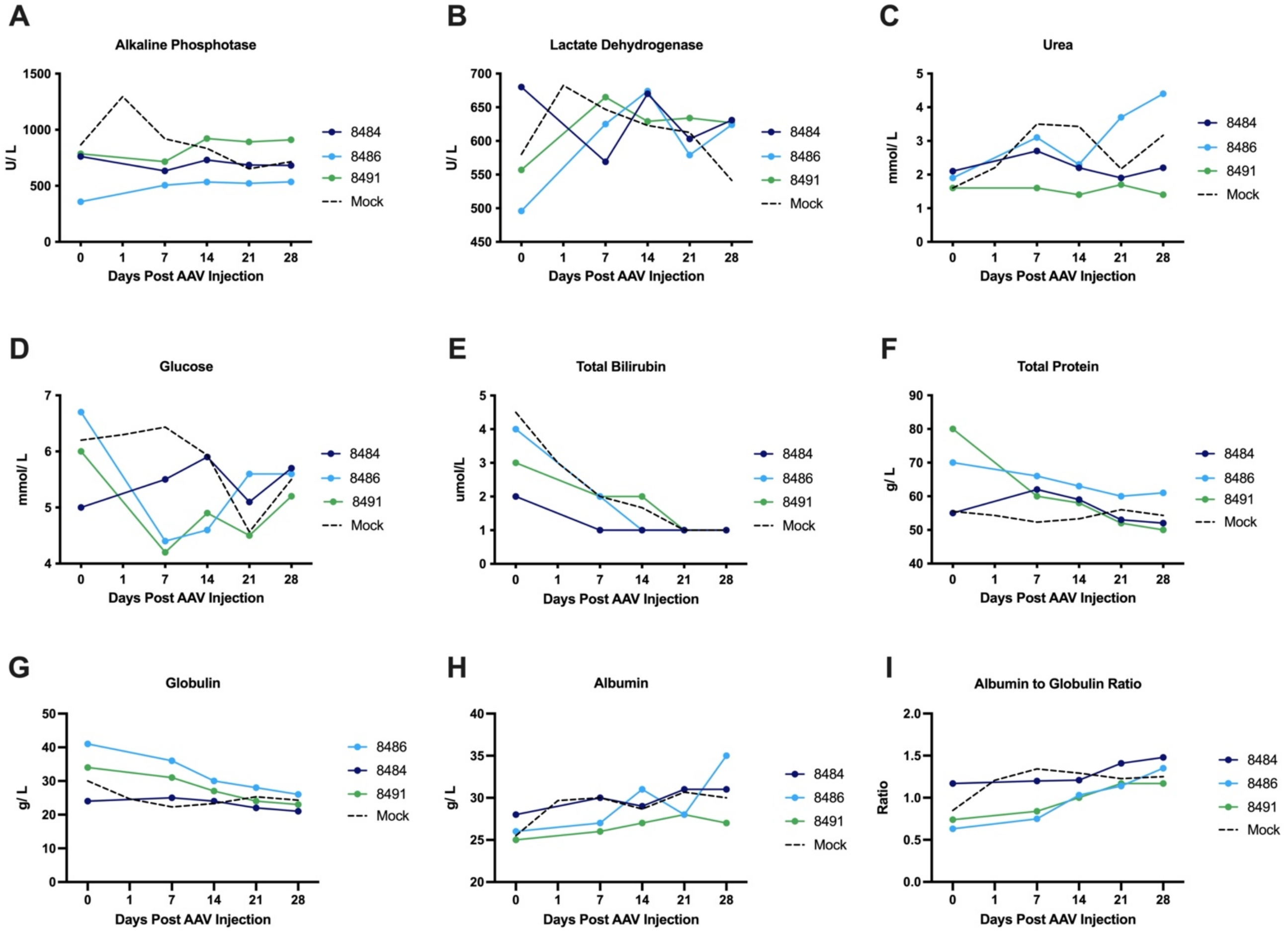

Supplemental Figure S6. Additional ovine clinical chemistry. Plasma concentrations of (A) alkaline phosphatase, (B) lactate dehydrogenase, (C) urea, (D) glucose, (E) bilirubin, (F) total protein, (G) globulin, (H) albumin and (I) the globulin to albu-min ratio.

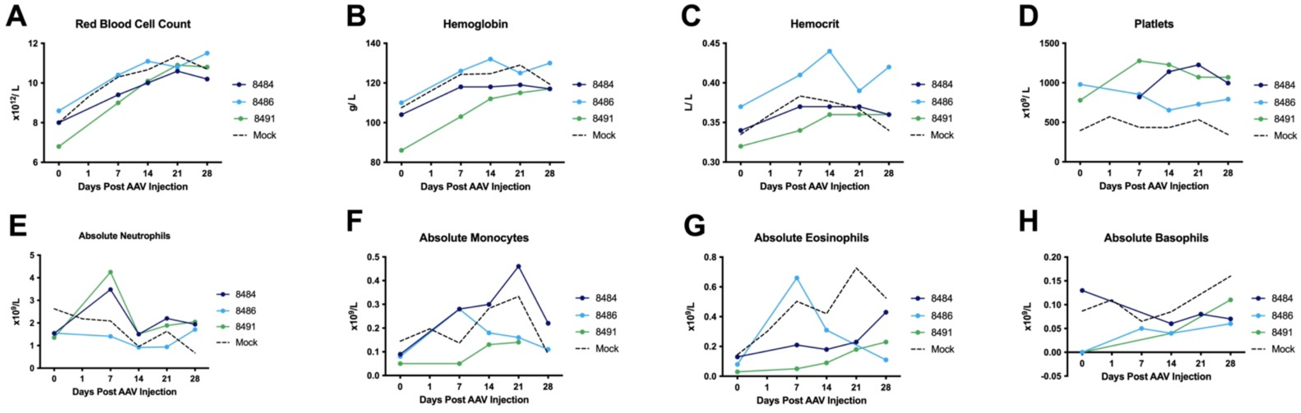

Supplemental Figure S7. Additional ovine hematology. (A) Red blood cell count, (B) hemoglobin concentration, (C) hemocrit concentration, (D) platelet, (E) neutrophil, (F) monocyte, (G) eosinophils and (H) basophil counts.

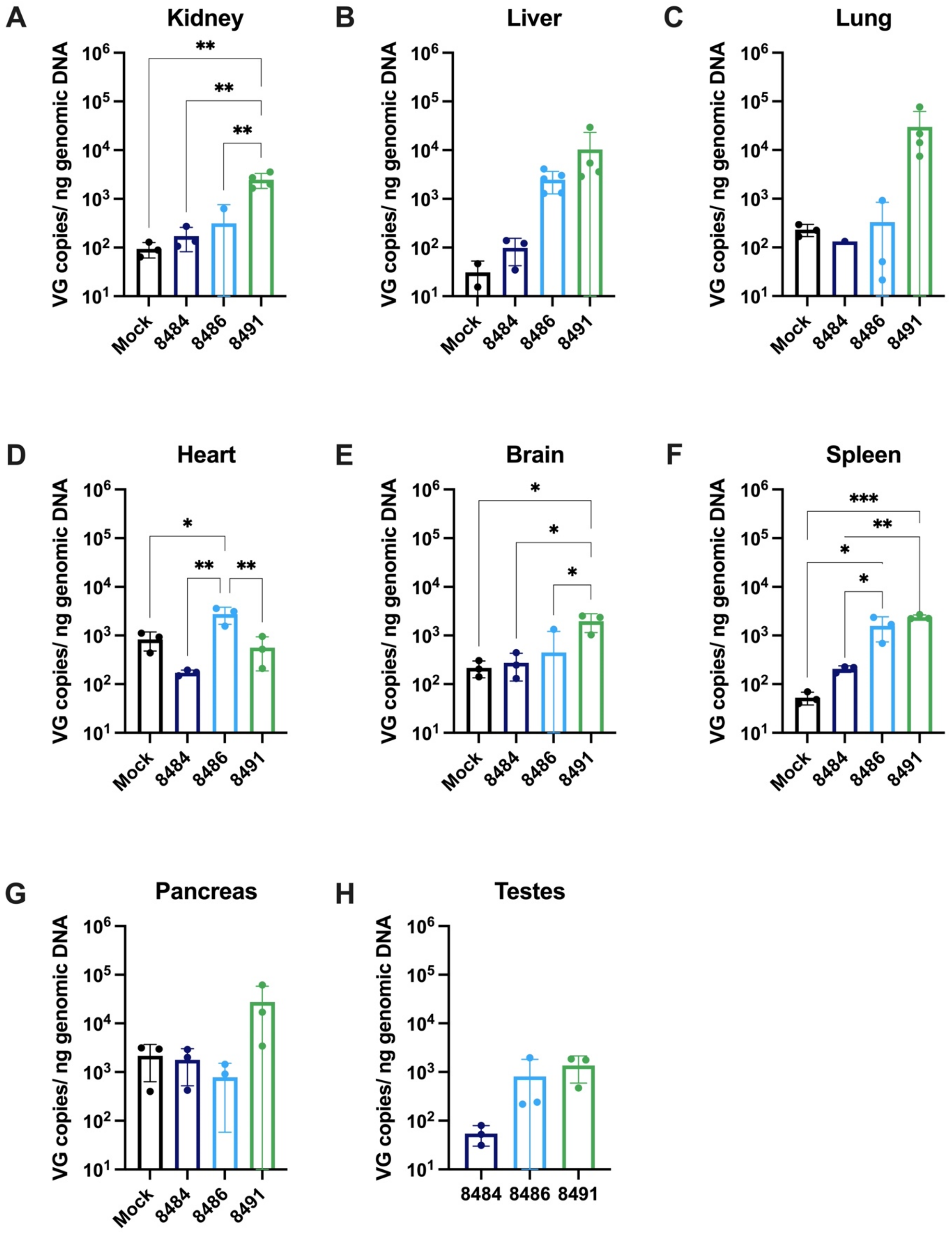

Supplemental Figure S8. Biodistribution of AAV in Ovine Tissues following IM administration of AAV6.2FF-31C2. AAV vec-tor genomes from (A) kidney, (B) liver, (C) lung, (D) heart, (E) brain and (F) spleen, (G) pancreas, (H) and testes tissue sam-ples were quantified by qPCR and shown as VG copies/ng genomic DNA. Data are represented as the mean ± standard devi-ation using a one-way ANOVA. *p<0.05, **p<0.01, ***p<0.001 and ****p<0.0001.

biomedicines

MDPI

Safety and tolerability of the adeno-associated virus vector, AAV6.2FF, expressing a monoclonal antibody in murine and ovine animal models

Supplementary Material

Table S1. Mouse H&E Grading

		ORGANS						
**COHORT**	**MOUSE ID**	**LUNG**	**HEART**	**BRAIN**	**KIDNEY**	**SPLEEN**	**MUSCLE**	**Liver**
Day 7-low	D7L-1	Normal. Apical blebs in terminal airways. Rare macrophages in alveoli.	Normal.	Normal.	Normal. Brown peri-renal fat.	Normal. Presence of PALS and B-cell follicles. EMH in red pulp.	Rare myofiber degeneration and rowing. Scattered plasma cells and macrophages in permysium.	Normal. Diffuse mild glycogen type vacuolation. Mild anisokaryosis and binucleation. Rare EMH in portal spaces.
	D7L-2	Normal. Apical blebs in terminal airways. Rare macrophages in alveoli.	Normal.	Normal.	Normal. Brown peri-renal fat.	Normal. Presence of PALS and B-cell follicles. EMH in red pulp.	Normal.	Normal. Diffuse mild glycogen type vacuolation. Mild anisokaryosis and binucleation. Rare EMH in portal spaces.
	D7L-3	Normal. Apical blebs in terminal airways. Rare macrophages in alveoli. Rare megakaryocytes in septa.	Normal.	Normal.	Normal. Brown peri-renal fat.	Normal. Presence of PALS and B-cell follicles. EMH in red pulp.	Normal.	Normal. Diffuse mild glycogen type vacuolation. Mild anisokaryosis and binucleation.
	D7L-4	Normal. Apical blebs in terminal airways. Rare macrophages in alveoli. Rare megakaryocytes in septa. Lymphoid clusters in pleura, rare and multifocal.	Normal.	Normal.	Normal. Brown peri-renal fat.	Normal. Presence of PALS and B-cell follicles. EMH in red pulp.	Normal.	Normal. Diffuse mild glycogen type vacuolation. Mild anisokaryosis and binucleation. Rare EMH in portal spaces.

	D7L-5 (adrenal)	Normal.<br>Apical blebs in terminal airways. Rare macrophages in alveoli. Rare megakaryocytes in septa. Lymphoid clusters in pleura, rare and multifocal.	Normal.	Normal.	Normal. Brown peri-renal fat.	Normal. Presence of PALS and B-cell follicles. EMH in red pulp.	Rare, scattered macrophages and neutrophils in the endomysium (lots of crush artifact)	Normal. Diffuse mild glycogen type vacuolation. Mild anisokaryosis and binucleation. Rare lymphoid clusters in subendothelial position in a large supra-hepatic vessel.
	D7L-6	Normal.<br>Apical blebs in terminal airways. Rare macrophages in alveoli. Rare megakaryocytes in septa	Normal.	Normal.	Normal. Brown peri-renal fat. One focus of lymphocytes in the interstitium.	Normal. Presence of PALS and B-cell follicles. EMH in red pulp.	Normal.	Normal. Diffuse mild glycogen type vacuolation. Mild anisokaryosis and binucleation. Scattered lymphocytes and plasma cells in portal spaces (possibly some EMH).
Day 7-mid	D7M-1	Normal.<br>Apical blebs in terminal airways. Few to none macrophages in alveoli.	Normal. Rare lymphocytes in epicardium in coronary groove.	Normal.	Normal. Brown peri-renal fat. Rare tubules in the cortex have vacuolation in epithelial cells.	Normal. Presence of PALS and B-cell follicles. EMH in red pulp.	Normal.	Normal. Diffuse mild glycogen type vacuolation. Mild anisokaryosis and binucleation. Rare (< 5%) hepatocytes have lipid type vacuolations.
	D7M-2	Normal.<br>Apical blebs in terminal airways. Rare macrophages in alveoli.	Normal.	Normal.	Normal. Brown peri-renal fat. One focus of lymphocytes in the interstitium of medulla.	Normal. Presence of PALS and B-cell follicles. EMH in red pulp. Follicles are prominent and coalescing.	Normal. Very rare inflammatory cells in perimysium.	Normal. Diffuse mild glycogen type vacuolation. Mild anisokaryosis and binucleation. Rare (< 5%) hepatocytes have lipid type vacuolations. Rare foci of EMH.
	D7M-3	Normal.<br>Apical blebs in terminal airways. Rare macrophages in alveoli.	Normal. Mild epicardial mineralization.	Normal.	Normal. Brown peri-renal fat.	Normal. Presence of PALS and B-cell follicles. EMH in red pulp. Follicles are prominent and coalescing.	Normal.	Normal. Diffuse mild glycogen type vacuolation. Mild anisokaryosis and binucleation. Rare (< 5%) hepatocytes have lipid type vacuolations.
	D7M-4	Normal.<br>Apical blebs in terminal airways. Rare macrophages in alveoli.	Normal	Normal.	Normal. Brown peri-renal fat.	Normal. Presence of PALS and B-cell follicles. EMH in red pulp. Follicles are prominent and coalescing.	Normal.	Normal. Diffuse mild glycogen type vacuolation. Mild anisokaryosis and binucleation. Rare (< 5%) hepatocytes have lipid type vacuolations. Scattered lymphocytes and possibly EMH in portal spaces.
	D7M-5	Normal.	Normal	Normal.	Normal. Brown peri-renal fat.	Normal. Presence of PALS and B-	Rare, scattered macrophages and	Normal. Diffuse mild glycogen type vacuolation. Mild anisokaryosis and

	(has esophagus)	Apical blebs in terminal airways. Rare macrophages in alveoli. Scattered EMH in alveoli. Rare lymphocytes around larger bronchioles.				cell follicles. EMH in red pulp. Follicles are prominent and coalescing.	neutrophils in the endomysium.	binucleation. Scattered lymphocytes and possibly EMH in portal spaces. Lymphoid clusters close to biliary ducts.
	D7M-6	Normal. Apical blebs in terminal airways. Rare macrophages in alveoli. Scattered EMH in septa.	Normal	Normal.	Normal. Brown peri-renal fat.	Normal. Presence of PALS and B-cell follicles. EMH in red pulp. Follicles are prominent and coalescing.	Normal.	Normal. Diffuse mild glycogen type vacuolation. Mild anisokaryosis and binucleation.
Day 7-high	D7H-1	Normal. Apical blebs in terminal airways. Rare macrophages in alveoli. Scattered EMH in septa.	Normal	Normal.	Normal. Brown peri-renal fat.	Normal. Presence of PALS and B-cell follicles. EMH in red pulp. Follicles are prominent and coalescing.	Focally, scattered lymphocytes and neutrophils.	Normal. Diffuse mild glycogen type vacuolation. Mild anisokaryosis and binucleation.
	D7H-2	Normal. Apical blebs in terminal airways. Rare macrophages in alveoli. Scattered EMH in septa.	Normal	Normal.	Normal. Brown peri-renal fat.	Normal. Presence of PALS and B-cell follicles. EMH in red pulp. Follicles are prominent and coalescing.	Normal.	Normal. Diffuse mild glycogen type vacuolation. Mild anisokaryosis and binucleation.
	D7H-3	Normal. Apical blebs in terminal airways. Moderate macrophages in alveoli. Scattered EMH in septa.	Normal	Normal.	Normal. Brown peri-renal fat.	Normal. Presence of PALS and B-cell follicles. EMH in red pulp. Follicles are prominent and coalescing. Scattered lymphocytes in perisplenic fat.	Scattered macrophages, lymphocytes, and neutrophils in the endomysium of two areas. Inflammation appears associated with hair fragments.A few myofibers are basophilic and have central nuclei.	Normal. Diffuse mild glycogen type vacuolation. Mild anisokaryosis and binucleation.
	D7H-4	Normal. Apical blebs in terminal airways. Scattered to low macrophages in	Normal	Normal.	Normal. Brown peri-renal fat.	Normal. Presence of PALS and B-cell follicles. EMH in red pulp. Follicles are	Normal.	Normal. Diffuse mild glycogen type vacuolation. Mild anisokaryosis and binucleation. Scattered lymphocytes in larger portal tracts.

		alveoli. Scattered EMH in septa. Rare clusters of lymphocytes around smaller vessels.				prominent and coalescing. Scattered lymphocytes in perisplenic fat.		
	D7H-5	Normal.<br>Apical blebs in terminal airways. Moderate numbers of macrophages in alveoli. Scattered EMH in septa. Rare clusters of lymphocytes in subpleural position.	Normal	Normal.	Normal. Brown peri-renal fat.	Normal. Presence of PALS and B-cell follicles. EMH in red pulp	Normal.	Normal. Diffuse mild glycogen type vacuolation. Mild anisokaryosis and binucleation. Scattered clusters of macrophages with Mott's cells, in perhepatic fat (possible cross section of lymph node).
	D7H-6	Normal.<br>Apical blebs in terminal airways. Moderate numbers of macrophages in alveoli. Scattered EMH in septa	Normal	Normal.	Normal. Brown peri-renal fat.	Normal. Presence of PALS and B-cell follicles. EMH in red pulp	Scattered lymphocytes, macrophages and neutrophils in the endomysium, some edema (multifocal).	Normal. Diffuse mild glycogen type vacuolation. Mild anisokaryosis and binucleation.
Day 7-mock	D7MK-1	Normal. Very well perfused.<br>Apical blebs in terminal airways. Moderate numbers of macrophages in alveoli. Scattered EMH in septa.	Normal	Normal.	Normal. Brown peri-renal fat.	Normal. Presence of PALS. Very little B germinal centers. EMH in red pulp	Scattered lymphocytes, and edema in endomysium. Scattered hair shafts. A few degenerating myofibers.	Normal. Diffuse mild glycogen type vacuolation.Rare <5% of lipid vacuolation in hepatocytes. Mild anisokaryosis and binucleation.
	D7MK-2	Normal. Apical blebs in terminal airways. Moderate numbers of macrophages in alveoli. Scattered EMH in septa (presence of a tracheobronchial node).	Normal	Normal.	Normal. Brown peri-renal fat.	Normal. Presence of PALS. Very little B germinal centers. EMH in red pulp	Normal.	Normal. Diffuse mild glycogen type vacuolation.Rare <5% of lipid vacuolation in hepatocytes. Mild anisokaryosis and binucleation.
	D7MK-3	Normal. Apical blebs in terminal airways. Low numbers of macrophages in alveoli. Scattered EMH in septa.	Normal. Rare lymphocytes in the epicardium in the coronary groove.	Normal.	Normal. Brown peri-renal fat.	Normal. Presence of PALS. Very little B germinal centers. EMH in red pulp	Edema, focal in endomysium, with scattered hair shafts.	Normal. Diffuse mild glycogen type vacuolation. Mild anisokaryosis and binucleation. Rare lymphocytes and EMH in portal spaces.
	D7MK-4	Normal. Apical blebs in terminal airways.	Normal. One cluster of	Normal.	Normal. Brown peri-renal fat.	Normal. Presence of PALS. Very	Normal.	Normal. Diffuse mild glycogen type vacuolation. Mild anisokaryosis and

		Low numbers of macrophages in alveoli. Scattered EMH in septa.	lymphocytes in the epicardium in the coronary groove.			little B germinal centers. EMH in red pulp		binucleation. Rare lymphocytes and EMH in portal spaces.
	D7MK-5	Normal. Apical blebs in terminal airways. Moderate numbers of macrophages in alveoli. Scattered EMH in septa.	Normal.	Normal.	Normal. Brown peri-renal fat.	Normal. Presence of PALS. Very little B germinal centers. EMH in red pulp	Normal.	Normal. Diffuse mild glycogen type vacuolation. Mild anisokaryosis and binucleation. Rare lymphocytes and EMH in portal spaces.
	D7MK-6 (this one has adrenal+ ovary)	Normal. Apical blebs in terminal airways. Very rare numbers of macrophages in alveoli. Scattered EMH in septa.	Normal.	Normal.	Normal. Brown peri-renal fat.	Normal. Presence of PALS. Very little B germinal centers. EMH in red pulp	Normal.	Normal. Diffuse mild glycogen type vacuolation. Mild anisokaryosis and binucleation
Day 28-low	D28L-1	Normal. Apical blebs in terminal airways. Very rare numbers of macrophages in alveoli. Scattered EMH in septa. Very well perfused.	Normal.	Normal.	Normal. Brown peri-renal fat.	Normal. Presence of PALS. Very little B germinal centers. EMH in red pulp	Scattered lymphocytes in the endomysium.	Normal. Diffuse mild glycogen type vacuolation. Mild anisokaryosis and binucleation. A few EMH and lymphocytes in rare portal spaces.
	D28L-2 (one adrenal as well)	Normal. Apical blebs in terminal airways. Very rare numbers of macrophages in alveoli. Scattered EMH in septa. Very well perfused.	Normal.	Normal.	Normal. Brown peri-renal fat.	Normal. Presence of PALS. Very little B germinal centers. EMH in red pulp	Scattered lymphocytes in the endomysium.	Normal. Diffuse mild glycogen type vacuolation. Mild anisokaryosis and binucleation.
	D28L-3	Normal. Apical blebs in terminal airways. Rare numbers of macrophages in alveoli. Very well perfused.	Normal.	Normal.	Normal. Brown peri-renal fat.	Normal. Presence of PALS. Very little B germinal centers. EMH in red pulp	Multifocal fibrosis and accumulation of lymphocytes and plasma cells in the endomysium. Scattered myofibers are degenerated.	Normal. Diffuse mild glycogen type vacuolation. Mild anisokaryosis and binucleation.
	D28L-4 (pancreas)	Normal. Apical blebs in terminal airways. Rare numbers of macrophages in alveoli. Scattered EMH in septa. Rare	Normal.	Normal.	Normal. Brown peri-renal fat.	Normal. Presence of PALS. Very little B germinal centers. EMH in red pulp	Normal (very little present).	Normal. Diffuse mild glycogen type vacuolation. Mild anisokaryosis and binucleation.Rare EMH and lymphocytes in portal spaces.

		lymphocytes around a bronchiole.						
	D28L-5	Normal. Apical blebs in terminal airways. Rare numbers of macrophages in alveoli. Rare lymphocytes around a vessel.	Normal.	Normal.	Normal. Brown peri-renal fat.	Normal. Presence of PALS. Numerous B germinal centers. EMH in red pulp	A few foci with lymphocytes around rare myofibers.	Normal. Diffuse mild glycogen type vacuolation. Mild anisokaryosis and binucleation.Rare EMH and lymphocytes in portal spaces. Rare multifocal areas with microvescicular lipidosis.
	D28L-6	Normal. Apical blebs in terminal airways. Rare numbers of macrophages in alveoli. Rare lymphocytes around vessels.	Normal.	Normal.	Normal. Brown peri-renal fat.	Normal. Presence of PALS. Rare B germinal centers. EMH in red pulp	Numerous foci with lymphocytes and plasma cells around rare myofibers. The adjacent connective tissue contains lymphocytes, rare plasma cells and scattered neutrophils. Perineuritis is also observed.	Normal. Diffuse mild glycogen type vacuolation. Mild anisokaryosis and binucleation .Rare EMH and lymphocytes in portal spaces.
Day 28-mid	D28M-1	Normal. Apical blebs in terminal airways. Rare numbers of macrophages in alveoli. Rare multifocal EMH.	Normal.	Normal.	Normal. Brown peri-renal fat.	Normal. Presence of PALS. Numerous B germinal centers. EMH in red pulp	Normal.	Normal. Diffuse mild glycogen type vacuolation. Mild anisokaryosis and binucleation.
	D28M-2 (adrenal)	Normal. Apical blebs in terminal airways. Rare numbers of macrophages in alveoli. Rare multifocal EMH.	Normal.	Normal.	Normal. Brown peri-renal fat.	Normal. Presence of PALS. Few B germinal centers. EMH in red pulp	Presence of lymphocytes and plasma cells in the fat around the muscle, and in the endomysium surrounding scattered myofibers.	Normal. Diffuse mild glycogen type vacuolation. Mild anisokaryosis and binucleation.
	D28M-3	Normal. Apical blebs in terminal airways. Rare numbers of macrophages in alveoli. Rare multifocal EMH.	Normal.	Normal.	Normal. Brown peri-renal fat.	Normal. Presence of PALS. Numerous B germinal centers. EMH in red pulp	Presence of lymphocytes and plasma cells in endomysium surrounding scattered myofibers. Rare degenerate myofibers.	Normal. Diffuse mild glycogen type vacuolation. Mild anisokaryosis and binucleation. Rare lipid-type vacuolation (< 5%)
	D28M-4	Normal. Apical blebs in terminal airways.	Normal.	Normal.	Normal. Brown peri-renal fat.	Normal. Presence of PALS. Rare B	Presence of lymphocytes and	Normal. Diffuse mild glycogen type vacuolation. Mild anisokaryosis and

	(adrenal)	Rare numbers of macrophages in alveoli. Rare multifocal EMH.				germinal centers. EMH in red pulp	plasma cells in endomysium surrounding scattered myofibers. Inflammation extends in fat round the muscle.	binucleation. Rare lymphocytes and EMH in portal spaces.
	D28M-5	Normal. Apical blebs in terminal airways. Rare numbers of macrophages in alveoli. Rare multifocal EMH.	Normal.	Normal.	Normal. Brown peri-renal fat.	Normal. Presence of PALS. Rare B germinal centers. EMH in red pulp	Presence of lymphocytes and plasma cells in endomysium surrounding scattered myofibers.	Normal. Diffuse mild glycogen type vacuolation. Mild anisokaryosis and binucleation.
	D28M-6	Normal. Apical blebs in terminal airways. Rare numbers of macrophages in alveoli. Rare multifocal EMH.	Normal.	Normal.	Normal. Brown peri-renal fat.	Normal. Presence of PALS. Numerous B germinal centers. EMH in red pulp	Normal.	Normal. Diffuse mild glycogen type vacuolation. Mild anisokaryosis and binucleation. Rare lymphocytes and EMH in portal spaces.
Day 28-high	D28H-1	Normal. Apical blebs in terminal airways. Rare numbers of macrophages in alveoli. Rare multifocal EMH.	Normal.	Normal.	Normal. Brown peri-renal fat.	Normal. Presence of PALS. Numerous B germinal centers. EMH in red pulp	Presence of lymphocytes and macrophages in endomysium surrounding scattered myofibers. A few myofibers replaced by inflammatory cells.	Normal. Diffuse mild glycogen type vacuolation. Mild anisokaryosis and binucleation.
	D28H-2	Normal. Apical blebs in terminal airways. Rare numbers of macrophages in alveoli. Rare multifocal EMH.	Normal.	Normal.	Normal. Brown peri-renal fat.	Normal. Presence of PALS. Numerous B germinal centers. EMH in red pulp	Presence of lymphocytes and macrophages in endomysium surrounding scattered myofibers. A few myofibers pyknotic.	Normal. Diffuse mild glycogen type vacuolation. Mild anisokaryosis and binucleation.
	D28H-3	Normal. Apical blebs in terminal airways. Rare numbers of macrophages in alveoli. Rare multifocal EMH.	Normal.	Normal.	Normal. Brown peri-renal fat.	Normal. Presence of PALS. Rare B germinal centers. EMH in red pulp	Normal.	Normal. Diffuse mild glycogen type vacuolation. Mild anisokaryosis and binucleation. <5% of lipid vacuolation in centrilobular hepatocytes.
	D28H-4	Normal. Apical blebs in terminal airways. Rare numbers of	Normal.	Normal.	Normal. Brown peri-renal fat.	Normal. Presence of PALS. Numerous B	Presence of lymphocytes and macrophages in	Normal. Diffuse mild glycogen type vacuolation. Mild anisokaryosis and binucleation.

	(small intestine and adrenal)	macrophages in alveoli. Rare multifocal EMH.				germinal centers. EMH in red pulp	endomysium surrounding scattered myofibers. A few myofibers pyknotic.	
	D28H-5	Normal. Apical blebs in terminal airways. Rare numbers of macrophages in alveoli. Rare multifocal EMH.	Normal.	Normal.	Normal. Brown peri-renal fat. Rare lymphocytes and plasma cells in the intima of the pelvis.	Normal. Presence of PALS. Numerous B germinal centers. EMH in red pulp	Presence of lymphocytes and macrophages in endomysium surrounding numerous myofibers. A few myofibers pyknotic. (one of the most affected)	Normal. Diffuse mild glycogen type vacuolation. Mild anisokaryosis and binucleation. Rare lymphocytes and EMH in portal spaces.
	D28H-6	Normal. Apical blebs in terminal airways. Rare numbers of macrophages in alveoli. Rare multifocal EMH. Rare lymphocytes and plasma cells around small vessels and bronchioles.	Normal.	Normal.	Normal. Brown peri-renal fat.	Normal. Presence of PALS. Numerous B germinal centers. EMH in red pulp	Presence of lymphocytes and macrophages in endomysium surrounding numerous myofibers. A few myofibers pyknotic. (one of the most affected)	Normal. Diffuse mild glycogen type vacuolation. Mild anisokaryosis and binucleation. Rare lymphocytes and EMH in portal spaces.
Day 28-mock	D28MK-1 (no spleen)	Normal. Apical blebs in terminal airways. Rare numbers of macrophages in alveoli. Rare multifocal EMH.	Normal.	Normal.	Normal. Brown peri-renal fat.	No spleen	Normal.	Normal. Diffuse mild glycogen type vacuolation. Mild anisokaryosis and binucleation. <5% of lipid vacuolation in centrilobular hepatocytes.
	D28MK-2 (popliteal node and adrenal)	Normal. Apical blebs in terminal airways. Rare numbers of macrophages in alveoli. Rare multifocal EMH.	Normal.	Normal.	Normal. Brown peri-renal fat.	Normal. Presence of PALS. Rare B germinal centers. EMH in red pulp	A cluster of degenerated myofibers.	Normal. Diffuse mild glycogen type vacuolation. Mild anisokaryosis and binucleation. Rare EMH and lymphocytes in portal spaces. <5% of lipid vacuolation in centrilobular hepatocytes.
	D28MK-3	Normal. Apical blebs in terminal airways. Rare numbers of macrophages in alveoli. Rare multifocal EMH.	Normal.	Normal.	Normal. Brown peri-renal fat.	Normal. Presence of PALS. Rare B germinal centers. EMH in red pulp	Normal.	Normal. Diffuse mild glycogen type vacuolation. Mild anisokaryosis and binucleation. <5% of lipid vacuolation in centrilobular hepatocytes.
	D28MK-4	Normal. Apical blebs in terminal airways. Rare numbers of	Normal.	Normal.	Normal. Brown peri-renal fat.	Normal. Presence of PALS. Rare to no B germinal	Normal.	Normal. Diffuse mild glycogen type vacuolation. Mild anisokaryosis and

		macrophages in alveoli. Rare multifocal EMH.				centers. EMH in red pulp		binucleation. <5% of lipid vacuolation in centrilobular hepatocytes.
	D28MK-5 (esophagus)	Normal. Apical blebs in terminal airways. Rare numbers of macrophages in alveoli. Rare multifocal EMH.	Normal.	Normal.	Normal. Brown peri-renal fat.	Normal. Presence of PALS. Rare to no B germinal centers. EMH in red pulp	Normal.	Normal. Diffuse mild glycogen type vacuolation. Mild anisokaryosis and binucleationRare EMH and lymphocytes in portal spaces. One lobe shows hepatocytes filled with lipid type vacuoilations and numerous activated Kuppfer's cells.
	D28MK-6 (skin)	Normal. Apical blebs in terminal airways. Rare numbers of macrophages in alveoli. Rare multifocal EMH.	Normal.	Normal.	Normal. Brown peri-renal fat.	Normal. Presence of PALS. Rare to no B germinal centers. EMH in red pulp	Normal.	Normal. Diffuse mild glycogen type vacuolation. Mild anisokaryosis and binucleation. Rare EMH and lymphocytes in portal spaces
Day 56-low	D56L-1	Normal. Very little lung. Rare numbers of macrophages in alveoli.	Normal.	Normal.	Normal. Brown peri-renal fat.	Normal. Presence of PALS. Rare to no B germinal centers. EMH in red pulp	Presence of lymphocytes, macrophages, and scattered neutrophils in endomysium surrounding numerous myofibers. A few myofibers are replaced by the inflammation, and the inflammation extends to the perimysial connective tissue.	Normal. Diffuse mild glycogen type vacuolation. Mild anisokaryosis and binucleation. <5% lipid vacuolation in centrilobular areas.
	D56L-2	Normal. Apical blebs in terminal airways. Rare numbers of macrophages in alveoli. Rare multifocal EMH	Normal.	Normal.	Normal. Brown peri-renal fat. A few lymphocytes in medullary interstitium.	Normal. Presence of PALS. Rare to no B germinal centers. EMH in red pulp	Presence of lymphocytes and macrophages in endomysium surrounding numerous myofibers. A few myofibers myofibers are replaced.	Normal. Diffuse mild glycogen type vacuolation. Mild anisokaryosis and binucleation. . <5% lipid vacuolation in centrilobular areas.
	D56L-3	Normal. Apical blebs in terminal airways. Rare numbers of macrophages in alveoli. Rare multifocal EMH	Normal.	Normal.	Normal. Brown peri-renal fat.	Normal. Presence of PALS. Rare to no B germinal centers. EMH in red pulp	Presence of rare lymphocytes and macrophages in endomysium.	Normal. Diffuse mild glycogen type vacuolation. Mild anisokaryosis and binucleation. . <5-10% lipid vacuolation in centrilobular areas.

	D56L-4	Normal. Apical blebs in terminal airways. Rare numbers of macrophages in alveoli. Rare multifocal EMH	Normal.	Normal.	Normal. Brown peri-renal fat.	Normal. Presence of PALS. Rare to no B germinal centers. EMH in red pulp	Presence of lymphocytes and macrophages in endomysium.	Normal. Diffuse mild glycogen type vacuolation. Mild anisokaryosis and binucleation. .
	D56L-5	Normal. Apical blebs in terminal airways. Rare numbers of macrophages in alveoli. Rare multifocal EMH	Normal.	Normal.	Normal. Brown peri-renal fat.	Normal. Presence of PALS. Rare to no B germinal centers. EMH in red pulp	Normal. A few atrophied/degenerate fibers. (very little muscle section)	Normal. Diffuse mild glycogen type vacuolation. Mild anisokaryosis and binucleation. .
	D56L-6	Normal. Apical blebs in terminal airways. Rare numbers of macrophages in alveoli. Rare multifocal EMH	Normal.	Normal.	Normal. Brown peri-renal fat.	Normal. Presence of PALS. Rare to no B germinal centers. EMH in red pulp	Presence of rare lymphocytes and macrophages in endomysium. (very little muscle section)	Normal. Diffuse mild glycogen type vacuolation. Mild anisokaryosis and binucleation.
Day 56-mid	D56M-1	Normal. Apical blebs in terminal airways. Rare numbers of macrophages in alveoli. Rare multifocal EMH	Normal.	Normal.	Normal. Brown peri-renal fat.	Normal. Presence of PALS.Numerous B germinal centers. EMH in red pulp	Presence of rare lymphocytes and macrophages in endomysium. (very little muscle section)	Normal. Diffuse mild glycogen type vacuolation. Mild anisokaryosis and binucleation.
	D56M-2	Normal. Apical blebs in terminal airways. Rare numbers of macrophages in alveoli. Rare multifocal EMH	Normal.	Normal.	Normal. Brown peri-renal fat.	Normal. Presence of PALS.Numerous B germinal centers. EMH in red pulp	Presence of rare lymphocytes and macrophages in endomysium. (little muscle section)	Normal. Diffuse mild glycogen type vacuolation. Mild anisokaryosis and binucleation. <5% lipid in centrilobular position.
	D56M-3	Normal. Apical blebs in terminal airways. Rare numbers of macrophages in alveoli. Rare multifocal EMH	Normal.	Normal.	Normal. Brown peri-renal fat.	Normal. Presence of PALS. Only one B germinal centers. EMH in red pulp (very small spleen section)	Normal.	Normal. Diffuse mild glycogen type vacuolation. Mild anisokaryosis and binucleation
	D56M-4 (adrenal)	Normal. Apical blebs in terminal airways. Rare numbers of macrophages in alveoli. Rare multifocal EMH. Perhaps tracheobronchial node.	Normal.	Normal.	Normal. Brown peri-renal fat.	Normal. Presence of PALS. Rare B germinal centers. EMH in red pulp	Normal.	Normal. Diffuse mild glycogen type vacuolation. Mild anisokaryosis and binucleation. Rare EMH and lymphocytes in portal spaces.

	D56M-5	Normal. Apical blebs in terminal airways. Rare numbers of macrophages in alveoli. Rare multifocal EMH	Normal.	Normal.	Normal. Brown peri-renal fat.	Normal. Presence of PALS. Rare to no B germinal centers. EMH in red pulp	Presence of lymphocytes and macrophages in endomysium around myofibers.	Normal. Diffuse mild glycogen type vacuolation. Mild anisokaryosis and binucleation. Rare EMH and lymphocytes in portal spaces.
	D56M-6 (sections of esophagus)	Normal. Apical blebs in terminal airways. Rare numbers of macrophages in alveoli. Rare multifocal EMH	Normal.	Normal.	Normal. Brown peri-renal fat.	Normal. Presence of PALS. Rare to no B germinal centers. EMH in red pulp	Presence of lymphocytes and macrophages in endomysium around myofibers.	Normal. Diffuse mild glycogen type vacuolation. Mild anisokaryosis and binucleation. Rare EMH and lymphocytes in portal spaces.
Day 56-high	D56H-1	Normal. Apical blebs in terminal airways. Rare numbers of macrophages in alveoli. Rare multifocal EMH	Normal.	Normal.	Normal. Brown peri-renal fat.	Normal. Presence of PALS. Numerous B germinal centers. EMH in red pulp	Presence of lymphocytes and macrophages in endomysium around myofibers.	Normal. Diffuse mild glycogen type vacuolation. Mild anisokaryosis and binucleation
	D56H-2	Normal. Apical blebs in terminal airways. Rare numbers of macrophages in alveoli. Rare multifocal EMH	Normal.	Normal.	Normal. Brown peri-renal fat.	Normal. Presence of PALS. No B germinal centers. EMH in red pulp	Presence of rare, scattered lymphocytes and macrophages in endomysium around myofibers.	Normal. Diffuse mild glycogen type vacuolation. Mild anisokaryosis and binucleation
	D56H-3	Normal. Apical blebs in terminal airways. Rare numbers of macrophages in alveoli. Rare multifocal EMH. A few lymphocytes in perivascular position in larger vessels.	Normal. Rare lymphocytes in epicardium.	Normal.	Normal. Brown peri-renal fat.	Normal. Presence of PALS. No B germinal centers. EMH in red pulp	Presence of rare, scattered lymphocytes and macrophages in endomysium around myofibers.	Normal. Diffuse mild glycogen type vacuolation. Mild anisokaryosis and binucleation
	D56H-4 (esophagus)	Normal. Apical blebs in terminal airways. Rare numbers of macrophages in alveoli. Rare multifocal EMH.	Normal.	Normal.	Normal. Brown peri-renal fat.	Normal. Presence of PALS. Numerous B germinal centers. EMH in red pulp	Presence of lymphocytes and macrophages in endomysium around myofibers.	Normal. Diffuse mild glycogen type vacuolation. Mild anisokaryosis and binucleation
	D56H-5	Normal. Apical blebs in terminal airways. Rare numbers of macrophages in alveoli. Rare multifocal EMH	Normal.	Normal.	Normal. Brown peri-renal fat.	Normal. Presence of PALS. No B germinal centers. EMH in red pulp	Presence of rare, scattered lymphocytes and macrophages in endomysium around myofibers.	Normal. Diffuse mild glycogen type vacuolation. Mild anisokaryosis and binucleation

	D56H-6	Normal. Apical blebs in terminal airways. Rare numbers of macrophages in alveoli. Rare multifocal EMH	Normal.	Normal.	Normal. Brown peri-renal fat.	Normal. Presence of PALS. Numerous B germinal centers. EMH in red pulp	MUSCLE MISSING	Normal. Diffuse mild glycogen type vacuolation. Mild anisokaryosis and binucleation. EMH and scattered lymphocytes in portal spaces.
Day 56-mock	D56MK-1	Normal. Apical blebs in terminal airways. Rare numbers of macrophages in alveoli. Rare multifocal EMH	Normal.	Normal.	Normal. Brown peri-renal fat.	Normal. Presence of PALS. Rare to none B germinal centers. EMH in red pulp	Normal	Normal. Diffuse mild glycogen type vacuolation. Mild anisokaryosis and binucleation
	D56MK-2	Normal. Apical blebs in terminal airways. Rare numbers of macrophages in alveoli. Rare multifocal EMH	Normal.	Normal.	Normal. Brown peri-renal fat.	Normal. Presence of PALS. Rare to none B germinal centers. EMH in red pulp	Normal	Normal. Diffuse mild glycogen type vacuolation. Mild anisokaryosis and binucleation
	D56MK-3	Normal. Apical blebs in terminal airways. Rare numbers of macrophages in alveoli. Rare multifocal EMH. Sections of tracheobronchial lymph node, in fat around lungs.	Normal.	Normal.	Normal. Brown peri-renal fat.	Normal. Presence of PALS. Rare to none B germinal centers. EMH in red pulp	Normal	Normal. Diffuse mild glycogen type vacuolation. Mild anisokaryosis and binucleation
	D56MK-4	Normal. Apical blebs in terminal airways. Rare numbers of macrophages in alveoli. Rare multifocal EMH.	Normal.	Normal.	Normal. Brown peri-renal fat.	Normal. Presence of PALS. Rare to none B germinal centers. EMH in red pulp	Normal	Normal. Diffuse mild glycogen type vacuolation. Mild anisokaryosis and binucleation
	D56MK-5	Normal. Apical blebs in terminal airways. Rare numbers of macrophages in alveoli. Rare multifocal EMH.	Normal.	Normal.	Normal. Brown peri-renal fat.	Normal. Presence of PALS. Rare to none B germinal centers. EMH in red pulp	Normal	Normal. Diffuse mild glycogen type vacuolation. Mild anisokaryosis and binucleation
	D56MK-6	Normal. Apical blebs in terminal airways. Rare numbers of macrophages in	Normal.	Normal.	Normal. Brown peri-renal fat.	Normal. Presence of PALS. Rare to none B germinal centers. EMH in red pulp	Normal	Normal. Diffuse mild glycogen type vacuolation. Mild anisokaryosis and binucleation

		alveoli. Rare multifocal EMH.						

Interpretation

With the exception of the muscle, all tissues appeared to be normal and the changes reported in this table are incidental.

Lungs. Presence of extramedullary hematopoiesis (EMH), scattered macrophages, and lymphocytes and plasma cells in the perivascular and peribronchial position – at the extent seen here – are interpreted as an incidental finding . Lymphocytes are part of the mucosa-associated lymphoid tissue (very small in mice). EMH was present in the interstitium or in the alveolar septa, occasionally giving an increased cellularity to the sections. Lungs had also different levels of formalin perfusion, but this did not impair the assessment of the pulmonary tissues. In a few cases, mediastinal fat with sections of what were interpreted to be tracheobronchial or mediastinal lymph nodes were captures on section. These tissues had no lesions.

Heart. The heart occasionally showed rare mineralization, and presence of scattered lymphocytes in the epicardium. These were considered to be incidental findings. Inflammatory cells in the epicardium were not associated with tissue damage, and may be part of previous hematopoietic nests.

Brain. No lesions or incidental findings were identified.

Spleen. The spleen presented a normal architecture, with peri-arteriolar lymphoid sheaths, B-dependent follicles (with germinal centers), a marginal zone, and a red pulp. The red pulp contained numerous areas of EMH. The table differentiates between spleens with prominent germinal centers and those without, however this is a very subjective distinction, as the germinal centers may not be capture on plane, for every section.

Liver. The liver presented mild ill-defined vacuolation (possible glycogen or expansion of endoplasmic reticulum upon fixation). These are NOT pathological changes. In a few cases, lipid-type vacuolations (crisp, well defined vacuoles) were present mainly in centrilobular spaces, always accounting <5% of the total hepatocytes. Vacuolation in centrilobular (part 3 of the lobule) areas is often seen as consequence of hypoxia, in fact this part of the lobule receives the least oxygenated blood. This change, being so limited in extent, is likely clinically insignificant. One case (TX16-2) showed marked lipid vacuolation that affected only one lobe. Correlation with history and postmortem finding is needed. Occasional omental fat with sections consistent with a lymph node were captured on section. These tissues had no lesions.

Other organs. When present, other organs (esophagus, adrenal, popliteal node, mesenteric fat, mediastinal with node, ovaries) were mentioned. No lesions in these organs were observed. The only ovary showed corpora lutea and follicles at different stages of differentiation.

Table S2. Sheep H&E Grading

Sheep ID	SLIDE ID / LABEL	ORGANS in the slide	DESCRIPTION
SHEEP #8484	GI	Large intestine	Normal
		Small intestine	Normal
		Abomasum	Normal. Only pyloric region, with numerous mucosa-associated lymphoid follicle
		Section of smooth muscle	Normal. Section of smooth muscle, with no mucosa (tangential section)
	NODE NERVE (no lymph node present)	Nerve	Normal. Numerous sections.
		Striated muscle	A few sections adjacent to the nerve. Rare myofibers surrounded by lymphocytes.
	BRAIN	Brain	Normal.
	SPLEEN & LUNG	Spleen	Normal. Extramedullary hematopoiesis (EMH) in red pulp
		Lung	Normal. A few lymphocytes and rare plasma cells around bronchioles and adjacent vessels (bronchus-associated lymphoid tissue – BALT)
	TESTIS	Testis	Diffuse tubular hypoplasia and azoospermia (immature testis)
		Bladder	Normal
	THYMUS	Pancreas	Normal (multifocal areas of autolysis)
		Thymus	Normal
	SPLEEN & LIVER	Liver	Normal. Scattered lymphocytes and extramedullary hematopoiesis in portal spaces and around sections of centrilobular veins.
		Kidney	Normal. A few interstitial lymphocytes (< 5% of the area). Very rare glomeruli have proliferative parietal epithelium (< 1% of glomeruli).
	BONE MARROW	Bone marrow	Normal. 40-60% cellularity; 1:1 Erythroid to myeloid ratio; 5-6 megakaryocytes per high power fields.
	THYROID	Epididymis	Normal.
		Thyroid & Parathyroid	Normal.
		Adrenal	Normal.
		Lymph node	Normal.
	HEART	Heart	Rare lymphocytes and scattered neutrophils in perivascular position and epicardium (< 5% of the area).
	SKIN (with recut)	Skin	Normal.

	MUSCLE B	Striated muscle	Very rare lymphocytes around myofibers.
	MUSCLE	Striated muscle	Normal
SHEEP #8486	GI	Striated muscle	Scattered myofibers surrounded by lymphocytes and few plasma cells
		Small intestine	Normal. Mucosa-associated lymphoid tissue in lamina propria
		Large intestine	Normal
		Abomasum	Normal. Mucosa-associated lymphoid tissue in lamina propria
		Striated muscle	Scattered myofibers surrounded by lymphocytes and few plasma cells (one of the 2 slides most affected)
	ADRENAL	Lymph node	Normal
		Adrenal	Normal
	NODES	Lymph node	Normal. Rare germinal centers in cortex
	NERVE (no nerve)	Striated muscle	Scattered myofibers surrounded by lymphocytes and few plasma cells
	BRAIN	Brain	Normal
	SPLEEN & LUNG	Spleen	Normal. Numerous germinal centers. Extramedullary hematopoiesis in red pulp.
		Lung	Normal. A few lymphocytes and rare plasma cells around bronchioles and adjacent vessels (bronchus-associated lymphoid tissue – BALT)
	TESTIS	Bladder	Normal
		Epididymis	Normal
		Testis	Diffuse tubular hypoplasia and azoospermia (immature testis)
	THYMUS	Striated muscle	Scattered myofibers surrounded by lymphocytes and few plasma cells
		Thymus	Normal
		Pancreas	Normal (multifocal autolysis)
	KIDNEY & LIVER	Kidney	Normal. A few interstitial lymphocytes (< 5% of the area). Very rare glomeruli have proliferative parietal epithelium (< 1% of glomeruli).
		Liver	Normal. Scattered lymphocytes and extramedullary hematopoiesis in portal spaces and around sections of centrilobular veins.
	BONE MARROW	Bone marrow	Normal. 40-50% cellularity. 1:1 = Erythroid:Myeloid ratio; 2-4 megakaryocytes per high power field

	THYROID	Striated muscle	Normal
		Thyroid	Normal
		Esophagus	Normal
	HEART	Heart	Normal
	SKIN	Striated muscle (subcutaneous muscle)	Scattered myofibers surrounded by lymphocytes and few plasma cells (one of the 2 slides most affected)
		Skin	Normal
	MUSCLE B	Striated muscle	Normal
	MUSCLE	Striated muscle	Scattered myofibers surrounded by lymphocytes and few plasma cells
SHEEP #8491	GI	Small intestine	Normal. Section through very prominent mucosa-associated lymphoid tissue (Peyer's patches)
		Large intestine	Normal
		Abomasum	Normal
	TRACHEA (with recut)	Trachea	Normal
		Abomasum	Normal. Scattered patches of mucosa-associated lymphoid tissue
	P NODE	Lymph node	Normal. Few germinal center in the cortex.
		Muscle	Normal
	ADRENAL	Adrenal	Normal. Rare foci of extramedullary hematopoiesis.
		Nerve and ganglion	Normal
		Striated muscle	Normal
	NODE	Lymph node	Normal
		Esophagus	Normal
	BRAIN	Brain	Normal
	SPLEEN & LUNG	Spleen	Normal. Numerous germinal centers. Extramedullary hematopoiesis in red pulp.
		Lung	Normal. A few lymphocytes and rare plasma cells around bronchioles and adjacent vessels (bronchus-associated lymphoid tissue – BALT); edema in alveolar spaces (peri-mortem artifact)

	TESTIS	Testis and epididymis	Normal. Hypoplastic (immature) testis.
		Pampiniform plexus	Normal
		Bladder	Normal
	THYMUS	Thymus	Normal
		Pancreas	Normal (multifocal autolysis)
	KIDNEY & LIVER	Kidney	Normal. A few interstitial lymphocytes (< 5% of the area).
		Liver	Normal. Scattered lymphocytes and extramedullary hematopoiesis in portal spaces and around sections of centrilobular veins.
	BONE MARROW	Bone marrow	Normal. 60-70% cellularity. 1:1 = Erythroid:Myeloid ratio; 2-4 megakaryocytes per high power field
	THYROID	Pancreas	Normal (some autolysis)
		Thyroid and parathyroid	Normal
		Lymph node	Normal. Little to no germinal centers
	HEART	Heart	Normal. A focal area of subendocardial fibrosis, interpreted as insertion of valves
	SKIN	Skin	Normal
	MUSCLE B	Striated muscle	Normal
	MUSCLE	Striated muscle	Normal
